# Allelic variation of the rice blast resistance gene *Pid3* in cultivated rice worldwide

**DOI:** 10.1038/s41598-017-10617-2

**Published:** 2017-09-04

**Authors:** Qiming Lv, Zhiyuan Huang, Xiao Xu, Li Tang, Hai Liu, Chunchao Wang, Zhuangzhi Zhou, Yeyun Xin, Junjie Xing, Zhirong Peng, Xiaobing Li, Tianqing Zheng, Lihuang Zhu

**Affiliations:** 1 State Key Laboratory of Hybrid Rice, Hunan Hybrid Rice Research Center, Changsha, 410125 China; 2 State Key Laboratory of Plant Genomics and National Center for Plant Gene Research, Institute of Genetics and Developmental Biology, Chinese Academy of Sciences, Beijing, 100101 China; 3 Institute of Crop Sciences/National Key Facility for Crop Gene Resources and Genetic Improvement, Chinese Academy of Agricultural Sciences, Beijing, 100081 China

## Abstract

In this study, the re-sequencing data from 3,000 rice genomes project (3 K RGP) was used to analyze the allelic variation at the rice blast resistance (*R*) *Pid3* locus. A total of 40 haplotypes were identified based on 71 nucleotide polymorphic sites among 2621 *Pid3* homozygous alleles in the 3k genomes. *Pid3* alleles in most *japonica* rice accessions were pseudogenes due to premature stop mutations, while those in most *indica* rice accessions were identical to the functional haplotype Hap_6, which had a similar resistance spectrum as the previously reported *Pid3* gene. By sequencing and CAPS marker analyzing the *Pid3* alleles in widespread cultivars in China, we verified that Hap_6 had been widely deployed in *indica* rice breeding of China. Thus, we suggest that the priority for utilization of the *Pid3* locus in rice breeding should be on introducing the functional *Pid3* alleles into *japonica* rice cultivars and the functional alleles of non-Hap_6 haplotypes into *indica* rice cultivars for increasing genetic diversity.

## Introduction

Rice (*Oryza sativa* L.) is a staple food for nearly half of the world’s population. It also represents a model for functional genome research among the crop plants. Rice was the first crop plant to be fully sequenced^1^ and so far, has at least four different reference genomes in two subspecies (*Oryza sativa subsp*. *indica* and *Oryza*. *sativa subsp*. *japonica*)^2–4^. Moreover, a lot of rice cultivars within different subpopulations and wild relatives in different *Oryza* species have their own genome assemblies^5–7^. Recently, with the cost reduction of sequencing, more studies on rice have been undertaken for exploring allelic variants through next generation sequencing (NGS)^8–15^. Particularly, the 3,000 rice genomes project (3 K RGP) has completed re-sequencing a core collection of 3,000 rice cultivars from 89 countries with an average sequencing depth of 14×, from which a total of 18.9 million single nucleotide polymorphisms (SNPs) were discovered when compared to the reference genome of *Nipponbare*, providing a complete picture of the total genetic diversity in the *O*. *sativa* gene pool^16–18^. With these whole genome sequences and large amount of re-sequencing data in hand, works for comparative rice genome researches^5, 10, 19^ and genome-wide association studies (GWAS) on important traits, such as grain size, grain weight, flowering time, metabolites, disease resistance and abiotic stress tolerance have been widely conducted^8, 12, 15, 20–25^. However intensive studies on allelic functional and nonfunctional variations of a certain type of genes or a specific gene locus were rarely reported.

Rice blast, caused by the filamentous ascomycete *Magnaporthe oryzae* (*M*. *oryzae*), is the most devastating rice fungus disease worldwide. It has been proven that deployment of cultivars with resistance (*R*) genes is the most effective and eco-friendly approach for the control of rice blast^26^. To date, at least 69 rice blast *R* loci have been identified, of which 16 loci harboring more than 30 *R* genes/alleles have been cloned and functionally analyzed in detail^27–30^. It is important to note that almost all cloned rice blast *R* genes encode nucleotide-binding site leucine-rich repeats (NBS-LRR) proteins except for *Pid2* ^31^ and *pi21* ^32^; the former encodes a receptor-like kinase and the later a proline-rich protein. Likewise, a number of *NBS-LRR* genes cloned from maize, sorghum, and brachypodium were also proved being blast resistant in rice^30, 33, 34^. In recent years, a trend has become clear: a significant number of newly cloned rice blast *R* genes have finally been verified as being allelic to one of the previously cloned rice blast *R* genes, and fewer represent a new rice blast *R* locus^29, 35^. Considering that there are more than 400 *NBS–LRR* gene sequences identified in a rice genome, and that allelic rice blast *R* genes may confer distinct resistance spectra to *M*. *oryzae* isolates^27, 29^, we believe that allele mining of cloned rice blast *R* genes in rice germplasms would reveal more favorable *R* alleles for rice blast resistance breeding^13^.

However, the majority of the cloned rice blast *R* genes are clustered^28, 36, 37^ as most of *NBS-LRR* genes present in diverse multigene families^30^. Moreover, the clustered *NBS-LRR* genes usually fall into heterogeneous groups based on their structural similarity. For example, in the 76-kb chromosomal region containing the rice blast *R* gene *Pi9* locus, six tandemly arranged *NBS-LRR* type putative genes were identified. The identities among the six paralogs ranged from 63.8 to 98.6% and only the *Nbs2-Pi9* was proved to be the *Pi9* gene^37^. The other example is *Pi5*, whose blast resistance function is actually conferred by two *NBS-LRR* genes, *Pi5-1* and *Pi5-2* ^38^. The ~90-kb sequences of the *Pi5* locus are significantly diverged between resistant and susceptible rice cultivars; the susceptible cultivar *Nipponbare* completely lacks the corresponding allele of *Pi5-2* ^38^. Similar statuses were also found at other rice blast *R* loci, like *Pik* ^39^, *Pia* ^40^, *Pi37* ^41^, *Pb1* ^42^, and *Pit* ^43^. These duplicated sequences have diverged through accumulated mutation, which increase the complexity of *NBS-LRR* gene sequences. Therefore, it is difficult to identify alleles of cloned *NBS-LRR* type rice blast *R* genes through allele mining approach based on either traditional PCR or NGS data analyzing. However, at a few rice blast *R* loci, the structure of *NBS-LRR* genes are rather simple, with only single *NBS-LRR* gene. Allele mining at these loci is feasible.

The rice blast *R* gene *Pid3* was initially identified in the *indica* variety Digu by performing a genome-wide comparison of paired *NBS-LRR* genes and their pseudogene alleles between 93-11 (*indica*) and *Nipponbare* (*japonica*) on the premise of the verification of obvious different resistance of *indica* and *japonica* varieties to *M*. *oryzae* strains collected from south and north China^44^. Pid3 is a typical CC-NBS-LRR protein of 924 amino acids with no intron. Alleles in most *japonica* varieties were identified as pseudogenes due to the presence of a nonsense mutation at the nucleotide position 2209 starting from the translation initiation site; however, this pseudogene mutation did not occur in tested *indica* varieties, including African cultivated rice varieties and AA genome-containing wild rice species^44^. Then, a number of *Pid3* alleles or orthologs were cloned by map-based cloning^45^ and sequencing-based allele mining from *indica* and wild rice accessions, of which five had been verified to confer differential resistance spectra to a set of *M*. *oryzae* isolates^29, 46^. In this study, mainly based on the 3 K RGP sequencing data, a total of 40 haplotypes were identified according to 71 nucleotide polymorphic sites in 2621 *Pid3* homozygous alleles. Finally, by PCR-based allele mining and gene transformation, we disclosed a functional *Pid3* allele, which has been widely deployed in *indica* rice cultivars and especially in hybrid rice in China. With the above overview, we may propose different strategies in application of the functional *Pid3* alleles to *indic*a and *japonica* rice breeding.

## Results

### The nonsense mutation of *Pid3* alleles at the position 2209

**Figure 1:**
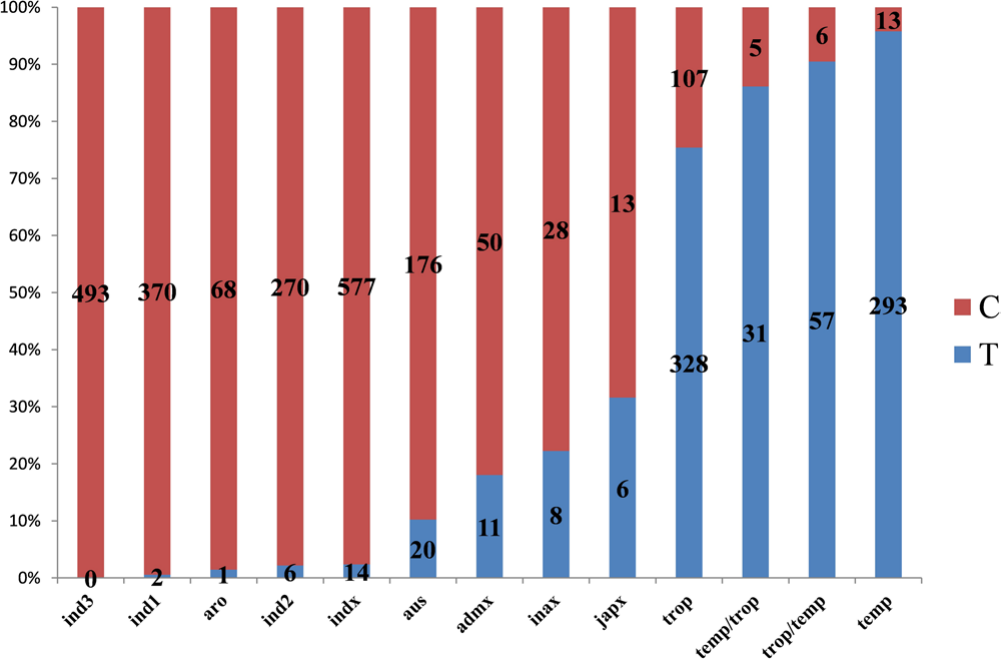
The nonsense mutation of *Pid3* alleles at the nucleotide position 2209. Blue “T” represents nonsense mutation. Ind1, ind2 and ind3 are three subgroups of *indica* rice, indx corresponds to other *indica* varieties, temp is temperate *japonica*, trop is tropical *japonica*, temp*/*trop and trop*/*temp are admixed temperate and tropical *japonica* varieties, japx is other *japonica* varieties, aus is aus, inax is admixed aus and *indica*, aro is aromatic, and admix is all other unassigned varieties^18^.

We previously revealed that *Pid3* alleles in 29 out of 32 *japonica* varieties were identified as pseudogenes due to the presence of a nonsense mutation (CAG to TAG) at the nucleotide position 2209, whereas none of the varieties in 32 *indica* collection contained this mutation^44^. To figure out the distribution of nonsense mutation of *Pid3* alleles in the 3 K RGP sequencing data^18^, we checked the corresponding position 13055819 on chromosome 6, where “G” represents “C” and “A” represents “T”, since *Pid3* coding sequence is on the “-” chain of the sequencing data. A total of 2953 *Pid3* alleles at this position were identified, of which 22 out of 1732 *indica*, 715 out of 859 *japonica* and 40 out of 362 other rice accessions were “A”, indicating *Pid3* alleles in most *japonica* and scarcely in *indica* rice accessions were nonfunctional due to the nonsense mutation at the position 2209. The detailed information was shown in Fig. 1. To verify the general survey based on the sequencing data, we used the CAPS marker^44^ to test nearly 300 varieties, including 149 widely cultivated *japonica* varieties in north China and 140 *indica* varieties, most of which were backbone parents of hybrid rice cultivars widely used in China. We found the nonsense mutation in 91.9% of the 149 *japonica* cultivars but neither of the 140 *indica* cultivars. The details for these cultivars were listed in Supplementary Table S1. Both of the results further confirmed our previous report that the pseudogenization of *Pid3* has prevailed in *japonica*.

### Nucleotide polymorphisms of *Pid3* alleles

The coding sequence of *Pid3* is located in a region (13055253-13058027) on chromosome 6 according to the *Nipponbare* genome in the 3 K RGP sequencing data. In this region, only 16 *Pid3* alleles showed obvious InDel polymorphisms at one singleton nucleotide site, which is an 8-bp (ATATATTC) insertion at the position 13055566, corresponding to the nucleotide position 2461 starting from the translation initiation site of *Pid3* gene. Of the 16 *Pid3* allelic loci, three showed heterozygous insertions, and all alleles were from *indica* subpopulation excepting one allele from *japonica* (Supplementary Table S2). After eliminating alleles with heterozygous sequences and ambiguous single site deletion probably caused by insufficient sequencing coverage, we have obtained a total of 2621 *Pid3* alleles for subsequent analyses.

**Figure 2:**
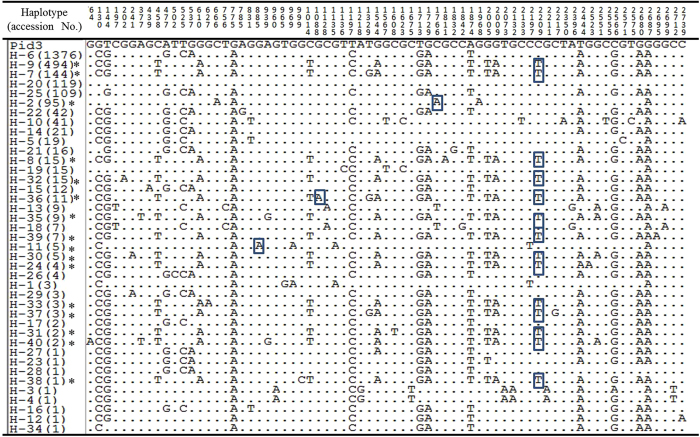
Summary of DNA variations in the 2775-bp coding region of 2621 *Pid3* alleles. Numbers in brackets represent rice accessions belonging to specific haplotypes. Site 1 corresponds to the first position of the start codon. Dots represent nucleotide variants identical to the *Pid3* sequence in Digu. The boxed nucleotide is the premature mutation.

In total, 71 nucleotide polymorphic sites were detected in the 2775-bp coding region of these 2621 *Pid3* alleles (Fig. 2). It was consistent to our previous finding^29^ that the *Pid3* gene processed lower nucleotide polymorphism (π = 0.00255 in this study), belonging to the conserved type of plant *NBS-LRR* class *R* genes^47^. Among the surveyed six subpopulations, *indica* has the lowest nucleotide polymorphism (π = 0.00102), while aromatic basmati/sadri (aro) group has the highest (π = 0.00265). The values of Tajima’s D were mostly negative but did not significantly deviate from the neutral model (Table 1). To figure out detailed information, three domains including CC, NBS and LRR were defined as those in our former study^29, 44^. Meanwhile, we assigned *Pid3-W5*, a functional *Pid3* ortholog cloned from *Oryza*. *rufipogon* ^29, 46^ as the reference, and calculated the Ka/Ks ratio between each *Pid3* haplotype and *Pid3-W*5 (Supplementary Table S3). On average, the nucleotide polymorphisms in NBS domain were comparable with those in LRR domain, but much higher than those in CC domain. However, most of the ratios of π_non_/π_syn_ and Ka/Ks in NBS domain were far less than 1, while they were much greater than 1 in LRR domain, indicating that the nucleotide polymorphisms in NBS domain were affected by purifying selection, while polymorphisms in LRR domain mainly affected by positive selection (Table 1 and Supplementary Table S3).

**Table 1:** Polymorphism, neutral test and haplotype analysis of *Pid3* alleles.

Population	Number	Region	S	π	Tajima’s D	π_non_	π_syn_	π_non_/π_syn_	Nhap	Hd
ALL	2621	Coding	71	0.00255	−0.47095	0.00222	0.00372	0.5968	40	0.680
		CC	6	0.00143	−0.61872	0.00117	0.00224	0.5223	8	0.275
		NBS	25	0.00254	−0.16943	0.00133	0.00699	0.1903	22	0.615
		LRR	28	0.00251	−0.60729	0.00317	0.00051	6.2157	20	0.579
Indica	1544	Coding	44	0.00102	−1.25381	0.00090	0.00143	0.6294	18	0.384
		CC	4	0.00148	−0.16974	0.00127	0.00213	0.5962	5	0.299
		NBS	14	0.00078	−1.09871	0.00034	0.00238	0.1429	11	0.247
		LRR	18	0.00093	−1.25942	0.00121	0.00005	24.2000	10	0.213
Japonica	784	Coding	54	0.00198	−0.74996	0.00200	0.00191	1.0471	19	0.614
		CC	6	0.00164	−0.70577	0.00123	0.00290	0.4241	7	0.275
		NBS	18	0.00163	−0.67529	0.00128	0.00294	0.4354	12	0.549
		LRR	21	0.00245	−0.36720	0.00292	0.00096	3.0417	9	0.343
Aus	163	Coding	39	0.00236	−0.14566	0.00198	0.00364	0.5440	10	0.706
		CC	2	0.00047	−0.84252	0.00042	0.00065	0.6462	3	0.140
		NBS	12	0.00202	0.07995	0.00056	0.00738	0.0759	8	0.656
		LRR	18	0.00300	−0.02360	0.00365	0.00093	3.9247	7	0.642
Inax	29	Coding	23	0.00178	−0.55900	0.00150	0.00270	0.5556	4	0.461
		CC	1	0.00023	−1.14923	0.00031	0	—	2	0.069
		NBS	8	0.00214	0.41815	0.00118	0.00565	0.2088	4	0.461
		LRR	10	0.00154	−1.16007	0.00203	0	—	3	0.431
Aro	56	Coding	30	0.00265	0.41230	0.00233	0.00372	0.6263	5	0.561
		CC	1	0.00012	−1.09119	0	0.00048	0.0000	2	0.036
		NBS	10	0.00127	−1.04112	0.00017	0.00528	0.0322	5	0.561
		LRR	14	0.00408	1.20787	0.00473	0.00202	2.3416	4	0.544
Admx	45	Coding	37	0.00240	−0.80080	0.00186	0.00422	0.4408	9	0.549
		CC	2	0.00071	−0.97392	0.00038	0.00171	0.2222	3	0.129
		NBS	14	0.00238	−0.61379	0.00108	0.00710	0.1521	7	0.536
		LRR	17	0.00267	−1.02375	0.00301	0.00156	1.9295	8	0.541

Indica, including ind1, ind2, ind3 and indx varieties; Japonica, including temp, trop, temp/trop, trop/temp and japx varieties; Aus, aus varieties; Inax, admixed aus and indica varieties; Aro, aromatic varieties; Admx, all other unassigned varieties. S, number of segregating sites; π, nucleotide diversity; π_non_, average nonsynonymous site diversity; π_syn_, average synonymous site diversity; π_non_/π_syn_, ratio of nonsynonymous site diversity over synonymous site diversity; Nhap, number of haplotype; Hd, haplotype diversity; *Statistical significance P < 0.05.

### Haplotype analysis of *Pid3* alleles

Based on the 71 nucleotide polymorphic sites, a total of 40 haplotypes of *Pid3* alleles were identified (Fig. 2 and Table 1). The average haplotype diversity (hd) of the *Pid3* coding region was 0.680. The aus/boro subpopulation had the highest hd (0.706), while the *indica* subpopulation has the lowest hd (0.384). The number of rice accessions in each haplotype varied significantly; Hap_6 was the largest haplotype shared by as many as 1376 rice accessions, whereas, in contrast to Hap_6, there were nine haplotypes each was carried by only one accession (Fig. 2). Besides the 14 haplotypes that had the premature stop codon at the above mentioned nucleotide position 2209, Hap_11, Hap_36 and Hap_2 were newly identified pseudogenization types with a premature stop codon at nucleotide position 885, 1088 and 1766, respectively. It is noteworthy that Hap_36 owned both of the premature stop codons at 1088 and 2209 simultaneously.

**Figure 3:**
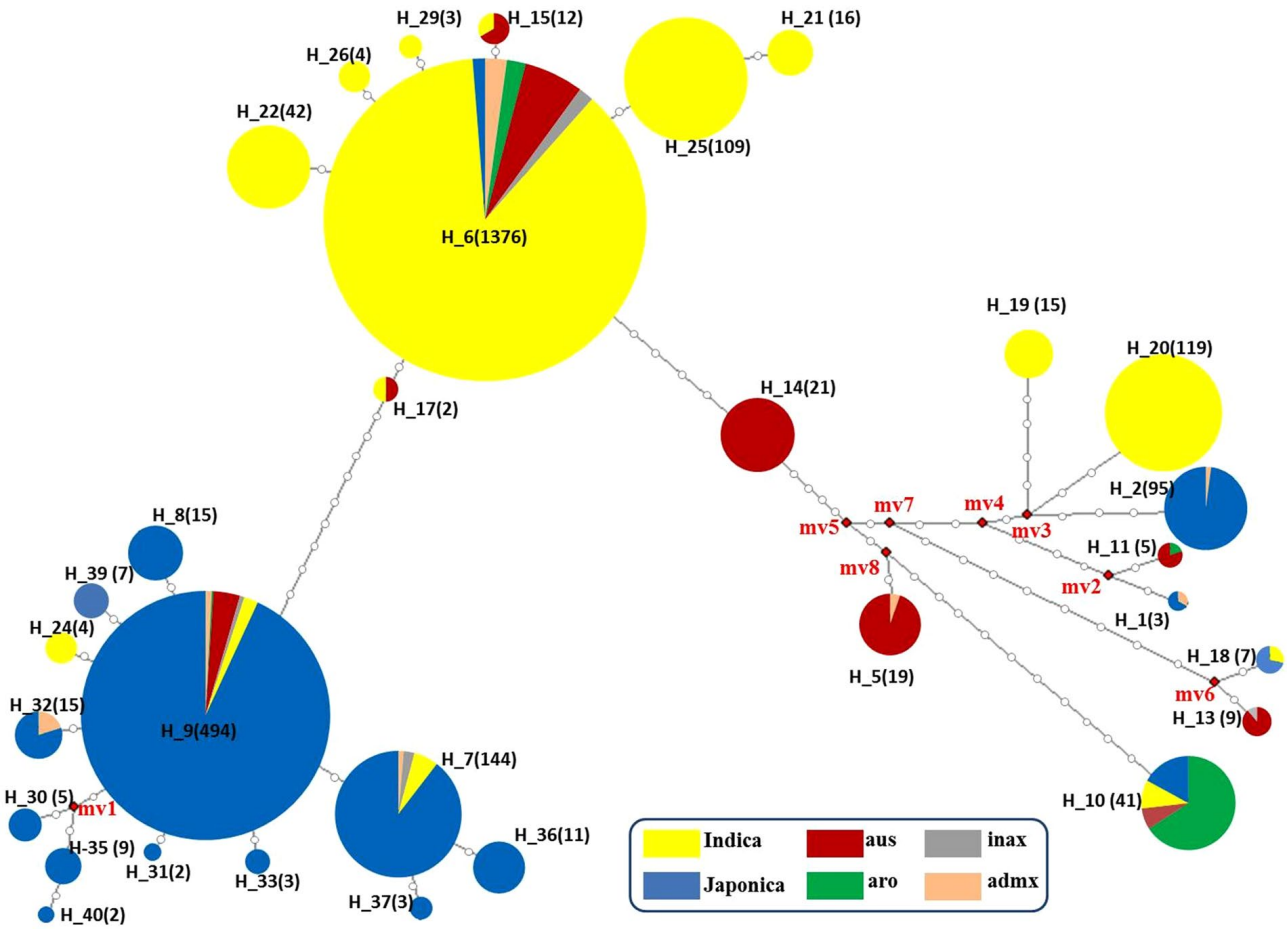
The flowchart of 31 haplotypes of *Pid3*. Each colored circle represents a unique haplotype. The size of the circle corresponds to the frequency of each haplotype. Each dot on a solid line represents one SNP between two haplotypes.

A haplotype flowchart was constructed to describe the evolutionary relationships and mutational steps of these 31 haplotypes, which were identified in at least two rice accessions (Fig. 3). Meanwhile, the components of each haplotype were also taken into account. The flowchart analysis illustrated that the haplotypes of *Pid3* could be roughly divided into three groups. Group I contains Hap_9 and twelve other haplotypes, in which most carriers are *japonica* accessions. All of these haplotypes have premature stop codon at 2209. Accordingly, in Group II, the predominant haplotype is Hap_6 but most of its carriers are *indica* accessions. The nucleotide diversities within group I and group II are much lower, and there only exist a few SNPs, as compared with Hap_9 and Hap_6, respectively. The remaining haplotypes belong to group III, in which no predominant haplotype exists, and the carriers of this group are diverse. It is notable that although Hap_2, shared mostly by *japonica* accessions, is not in group I, it is still a pseudogene due to the premature stop codon at 1766 as mentioned above. Indeed, out of the 95 *Pid3* alleles belonging to Hap_2, 86 were identified from tropical *japonica*. Moreover, the *Pid3* haplotypes from tropical and temperate *japonica* are significantly different (Supplementary Figure S1). Ten haplotypes and eleven haplotypes were identified from 287 temperate *japonica* and 392 tropical *japonica* rice accessions, respectively, and only five haplotypes were found in both subgroups. *Pid3* alleles in most temperate *japonica* belong to Hap_9, whereas Hap_9, Hap_7 and Hap_2 were mainly shared by tropical *japonica* rice accessions (Supplementary Figure S1).

In a previous study, we completely cloned 30 *Pid3* orthologs from 17 wild rice and 10 cultivated rice accessions by allele mining (wild rice accessions W1, W11, W13 were heterozygous)^29^. By comparing the sequences of these 30 orthologs with the above identified 40 haplotypes, we found that, out of the 10 *Pid3* orthologs cloned from cultivated rice, eight were identical to the newly discovered haplotypes (*Pid3-I1* = Hap_14, *Pid3-I2 = *Hap_6, *Pid3-I3* = Hap_20, *Pid3-I4 *= Hap_21, *Pid3-J1/J2* = Hap_9, *Pid3-J3/J5* = Hap_8). Meanwhile, out of the 20 *Pid3* orthologs from wild rice accessions, only four were identical to those haplotypes (*Pid3-W5* = Hap_13, *Pid3-W11-1* = Hap_9, *Pid3-W12* = Hap_6, *Pid3-W15* = Hap_18) (Supplementary Table S4). For the 10 cultivated rice orthologs, a total of 26 SNPs were identified, of which only the SNPs at the position 46 and 994 were not included by the above identified 71 SNPs. In the 20 *Pid3* orthologs from wild rice, a total of 101 SNPs were characterized, of which only 35 could be included by the above described 40 haplotypes (Supplementary Table S4).

**Figure 4:**
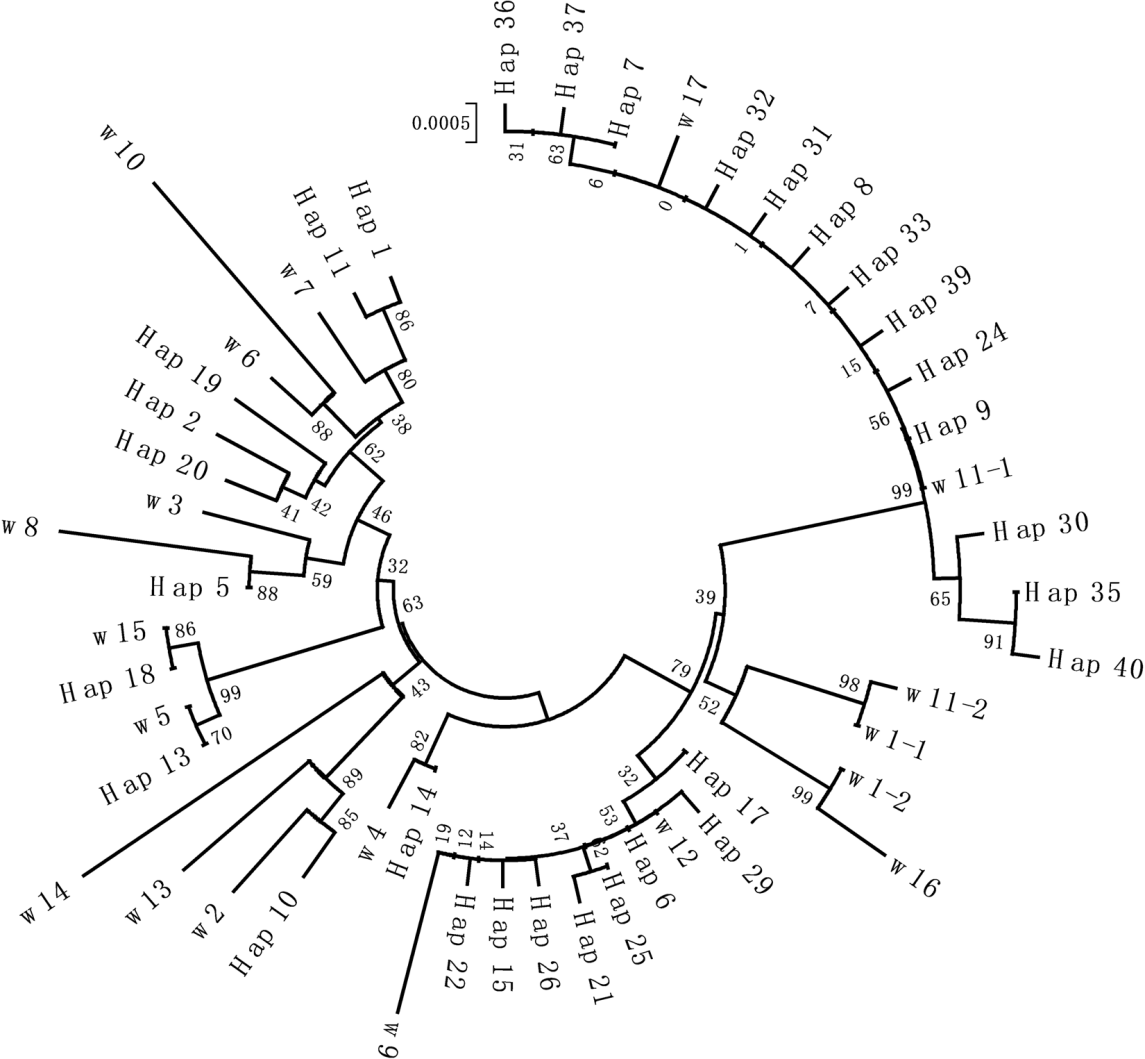
Phylogenetic analysis of *Pid3* haplotypes. The phylogenetic tree was generated by MEGA5 software using neighbor joining method, with the numbers associated with the interior branches indicating bootstrap values (1000 replications). The scale shows nucleotide substitutions per site.

The closest wild relatives of *O*. *sativa* are *O*. *nivara* and *O*. *rufipogon*, although which of them is the immediate progenitor of the cultivated rice remains controversial^9^. To investigate the domesticated history of *Pid3*, the 40 cultivated and 20 wild rice haplotypes were aligned (Fig. 4). It could be inferred that the haplotypes Hap_6 and Hap_9 were the ancestral types in cultivated rice, as they existed in all six cultivated rice subpopulations (Fig. 3) and two wild rice accessions, W12 and W11 (Fig. 4). In addition, they could be domesticated independently from different wild rice accessions, and the other haplotypes in group I and group II mentioned above might originate from Hap_9 and Hap_6, respectively. The haplotypes in group III might originate from a third type of wild rice accessions, because these haplotypes were much different from Hap_6 and Hap_9, and most of them were similar to those from wild rice accessions.

### Analysis of predicted Pid3 proteins

**Figure 5:**
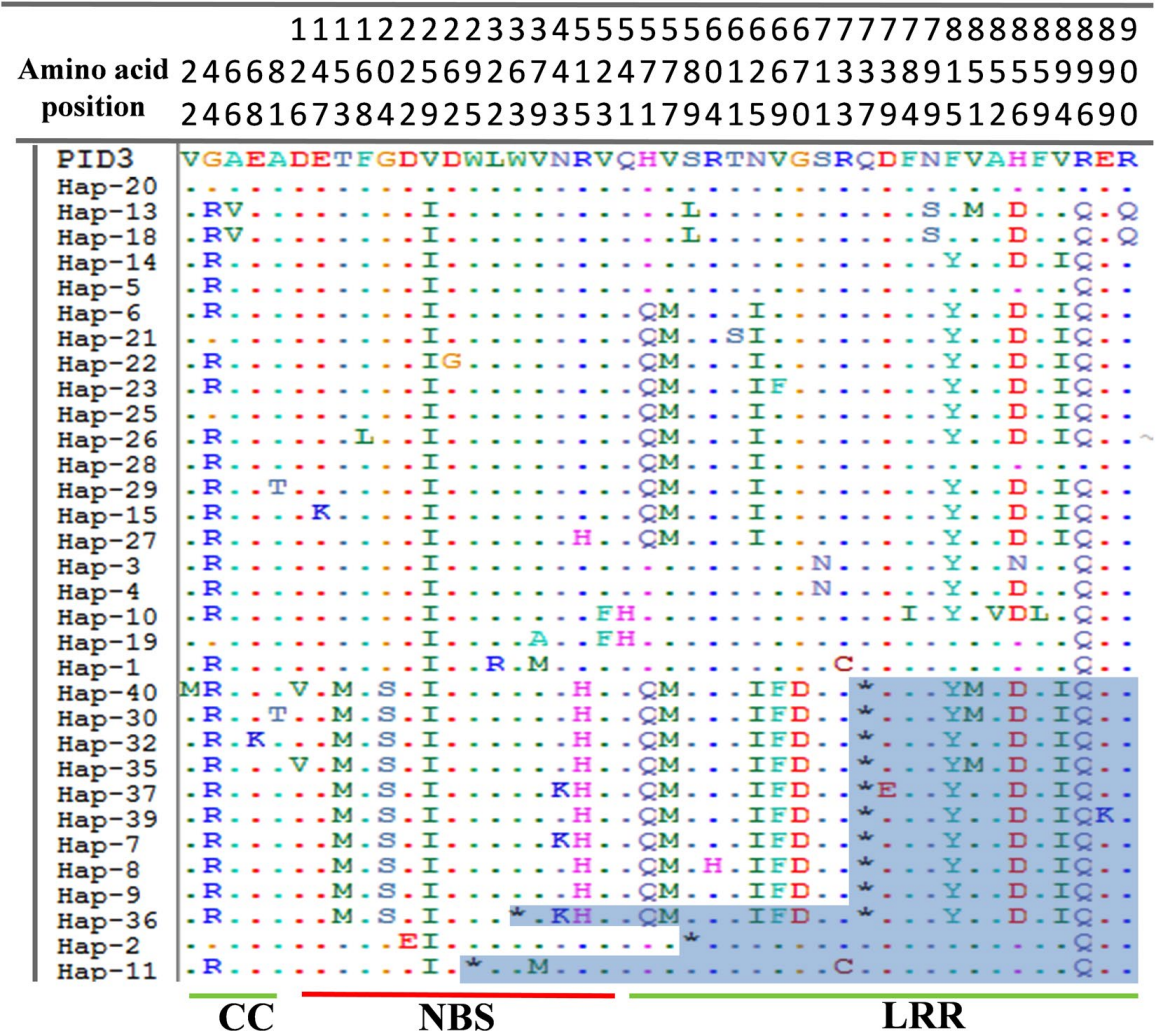
Amino acid variation of Pid3 in 40 haplotypes. “.” represents the same base as Pid3 in Digu; “*” represents terminators.

A total of 44 amino acid variations caused by the 71 nucleotide polymorphic sites, leads to 32 different predicted proteins (the original *Pid3* haplotype Hap_9 = Hap_24 = Hap_31 = Hap_33 = Hap_38 and Hap_6 = Hap_12 = Hap_16 = Hap_17 = Hap_34). Of them, 20 encode complete CC-NBS-LRR proteins with 924 amino acids, and 12 show premature transcription termination at the position 295, 363, 589 and 737, respectively (Fig. 5). Most predicted proteins encoded by *Pid3* alleles are different from Pid3 itself at nine positions, including 44, 259, 571, 577, 625, 815, 856, 894 and 896. It is noteworthy that besides the premature site between full length and truncated proteins at the position 737, there are five other completely different sites (153, 204, 515, 669 and 670) among them (Fig. 5), but this phenomenon was not found in other truncated proteins, which were premature at the position 295 and 589.

We also compared Pid3 protein sequences in different cultivated rice growing areas, including *indica/japonica* growing areas in East Asia and Southeast Asia, and *indica/japonica/aus/aro* growing areas in South Asia. The sequence comparison revealed that most amino acid variations were found in the LRR region, and *indica* subgroups had lower diversity in all three areas (Supplementary Figure S2). For *indica* and *japonica* subgroups in East Asia and Southeast Asia, *Pid3* haplotypes had no obvious difference. However, two stop codons at the position 589 and 737, five common variant amino acids, T153M, G204S, R515H in NBS domain, V669F, G670D in LRR region were found between *indica* and *japonica* subgroups in these two areas. We can infer that these variants are related to *indica -japonica* differentiation and play an important role in *Pid3* function. Most aus and aro rice cultivars were found in South Asia. In this area, except for the 15 aus cultivars belonging to the *japonica* predominant Hap_9, most of the remaining haplotypes were extremely similar to *indica* haplotypes.

### The *Pid3* ortholog (Hap_6) present a similar resistance spectrum as *Pid3* gene

**Figure 6:**
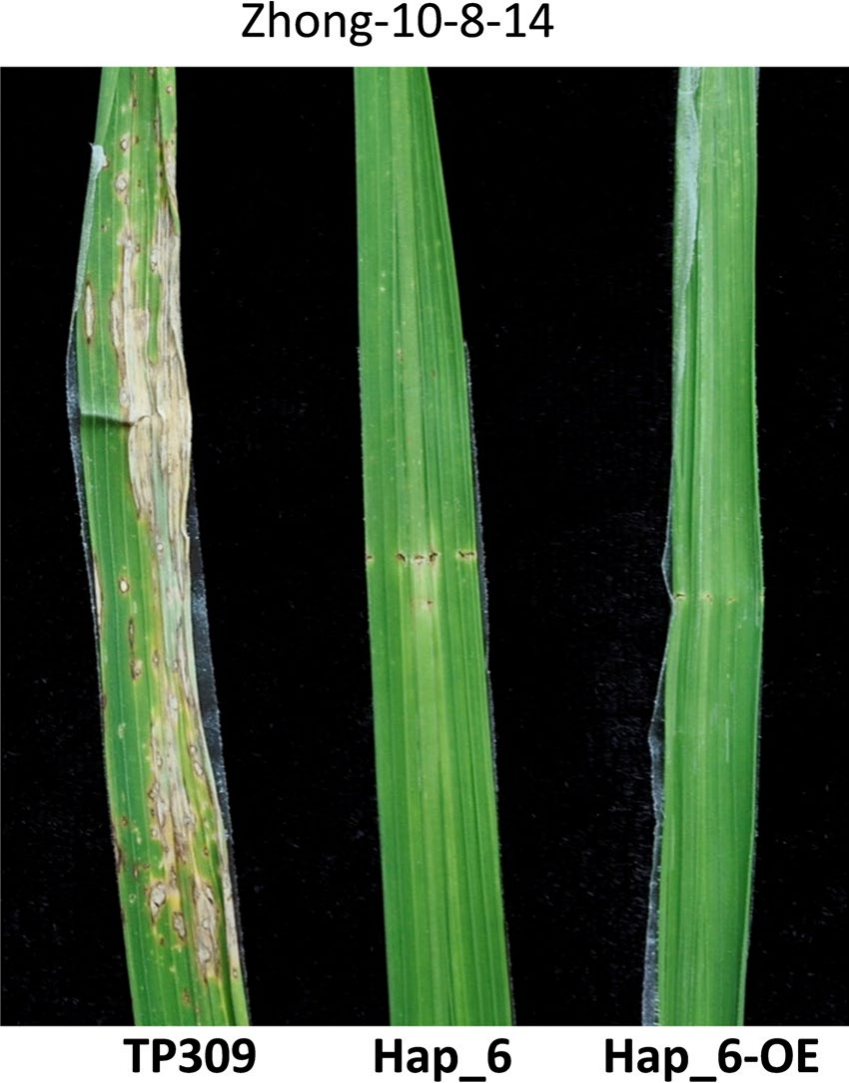
Complementation test and overexpression of haplotype Hap_6. The primary transgenic lines are inoculated by *M*. *oryzae* isolate Zhong-10-8-14. The susceptible variety TP309 is showed as control. Hap_6 represents *Pid3-I2* transgenic plant driven by itself promoter, and Hap_6-OE represents *Pid3-I2* transgenic plant driven by CAMV 35 S promoter.

In a previous study, we evaluated the resistance of 11 *Pid3* orthologs by rice genetic transformation and blast inoculation, and found that five *Pid3* orthologs were functional rice blast *R* genes, including *Pid3-I1*(Hap_14), *Pid3-I3* (Hap_20/*Pid3/Pi25*) from *indica* varieties and *Pid3-W3*, *Pid3-W4*, *Pid3-W5* (Hap_13/*Pid3-A4*) from wild rice accessions^29^. However, although it is the most popular haplotype in cultivated rice accessions, the rice blast resistance of the *Pid3* ortholog (Hap_6) had not been verified yet. In this study, *Pid3-I2* from the *indica* variety 93-11, a widely used inbred cultivar and backbone parent of hybrid rice in China, was chosen as the representative of Hap_6 for blast resistance testing. First, the entire 2775-bp coding region of *Pid3-I2* was inserted into the binary vector pZH01 under the (CAMV) 35 S promoter control and transformed into the susceptible rice variety TP309, which was the same recipient used for 11 *Pid3* orthologs in our previous study^29^. Next, we performed a genetic complementation test of *Pid3-I2* as previously described^44^. A 6236-bp 93-11’s DNA fragment, including the *Pid3-I2* coding region, 3010-bp upstream region, and 451-bp downstream region, was sub-cloned into the binary vector pMNDRBBin6, which was then introduced into TP309 as well. Finally, we obtained nine and eleven independent primary transgenic plants (T0) for these two constructs, respectively. All 20 transgene-positive plants were confirmed to be resistant to the *M*. *oryzae* isolate Zhong-10-8-14, which was the same isolate employed in our previous study (Fig. 6). Co-segregation of the transgene and the blast resistance was confirmed in selfed progenies (T1) of the two types of T0 lines, respectively. The results suggested that *Pid3-I2/*Hap_6 was indeed functional rice blast *R* gene. We then inoculated *Pid3* and *Pid3-I2/*Hap_6 homozygous T2 transgenic plants, respectively, with 125 *M*. *oryzae* isolates collected from China. The testing revealed that compared to the susceptible recipient TP309, *Pid3-I2/*Hap_6 transgenic lines conferred resistance to 28 isolates, with a resistance frequency 22.4%, which is the same as that of the *Pid3* transgenic plants (Supplementary Tables S5 and S6).

### Geographic distribution of the known functional *Pid3* alleles in cultivated rice accessions

At present, of the total of 42 haplotypes of *Pid3* identified in cultivated rice accessions, only four haplotypes, including Hap_6, Hap_13, Hap_14, and Hap_20 were confirmed to be functional in rice blast resistance. Of the remaining 38 haplotypes, 16 were identified as pseudogenes due to the presence of premature stop codons at different positions (Fig. 2), while the other 22 remained to be further elucidated. Based on the information of the 3 K RGP, the worldwide geographic distributions of the three types of *Pid3* haplotypes were presented in Table 2. Obviously, Hap_6 is the most common haplotype with the widest geographic distribution in the *indica*-cultivated area, whereas in most *japonica*-cultivated area, such as Japan, South Korea and Europe, functional haplotypes of *Pid3* are almost nonexistent. In Southeast Asia countries, such as Thailand, Vietnam, Cambodia and Myanmar, Hap_20 distributes widely, while Hap_14 is only found in four South Asia countries: India, Pakistan, Nepal and Bangladesh. Finally, Hap_13, the rarest functional haplotype in cultivated rice accessions, is only found in China, India and Bangladesh. Although the number of haplotypes (whose functions have not been determined yet) is up to 22, these haplotypes are distributed scarcely in most countries and areas.

**Table 2:** Geographic distributions of three types of haplotypes of *Pid3*.

Continents	Countries/Regions	Total rice accessions	Functional haplotypes				Non-functional haplotypes	Function not determined haplotypes
			Hap_6	Hap_20	Hap_14	Hap_13		
Asia	China	439	265	13	0	2	112	47
	India	351	241	6	3	6	43	52
	Indonesia	210	112	9	0	0	84	5
	Philippines	195	110	3	0	0	72	10
	Bangladesh	142	80	0	12	1	2	47
	Thailand	131	77	28	0	0	19	7
	Laos	114	51	5	0	0	54	4
	Malaysia	63	25	4	0	0	19	15
	Myanmar	57	38	12	0	0	4	3
	Cambodia	51	28	17	0	0	3	3
	Japan	51	3	0	0	0	44	4
	Vietnam	47	30	7	0	0	7	3
	Sri Lanka	45	34	0	0	0	3	8
	Nepal	43	31	0	2	0	6	4
	Taiwan	34	22	0	0	0	12	0
	South Korea	32	5	0	0	0	27	0
	Pakistan	29	11	0	4	0	4	10
	Bhutan	16	6	0	0	0	9	1
Africa	Madagascar	66	38	0	0	0	27	1
	Senegal	22	14	1	0	0	1	6
	Ivory Coast	21	5	0	0	0	15	1
	Sierra Leone	18	6	0	0	0	3	9
	Liberia	12	4	1	0	0	7	0
	Nigeria	12	8	0	0	0	3	1
Europe	Italy	37	2	0	0	0	35	0
	Portugal	22	0	1	0	0	21	0
	France	10	1	0	0	0	9	0
	Spain	10	0	0	0	0	10	0
South America	Colombia	24	12	2	0	0	8	2
	Brazil	22	8	0	0	0	10	4
	Argentina	11	1	0	0	0	10	0
North America	United States	47	3	0	0	0	43	1
Oceania	Australia	13	4	0	0	0	9	0

Only county/region containing more than 10 rice accessions were included; Non-functional haplotypes, including Hap_2, Hap_7, Hap_8, Hap_9, Hap_11, Hap_24, Hap_30, Hap_31, Hap_32, Hap_33, Hap_35, Hap_36, Hap_37, Hap_39, Hap_40; Function not determined haplotypes, including Hap_1, Hap_5, Hap_10, Hap_15, Hap_17, Hap_18, Hap_19, Hap_21, Hap_22, Hap_25, Hap_26, Hap_29.

### Hap_6 has been widely employed in hybrid rice breeding in China

To investigate the distribution of the known functional *Pid3* haplotypes in cultivated rice varieties in China, we first sequenced the respective allelic *Pid3* coding regions of the 12 widely cultivated *japonica* varieties in China, which would not contain the premature mutation at the position 2209 as testified by the CAPS marker (Supplementary Table S1). Sequence comparison confirmed that in these *japonica* varieties *Pid3* alleles were identical to Hap_6, suggesting that Hap_6 of *Pid3* might be introduced into minor *japonica* varieties by rice breeders. Next, we investigated whether *Pid3* alleles in widely cultivated *indica* varieties in China were identical to Hap_6 or not. We focused on backbone parental lines of hybrid rice varieties in China. We chose nine restorer lines (Minghui 63, Shuhui 527, Gui 99, Fuhui 838, Xianhui 207, Miyang 46, CDR22, IR24, Mianhui 725) and nine male sterility lines (II-32A, Zhenshan 97A, Jin23A, Tianfeng A, V20A, Gang 46 A, Peiai64S, Y58S, Guangzhan 63-4S) to fully sequence their *Pid3* alleles because they are most frequently used parents of hybrid rice in China (http://www.ricedata.cn/variety/). For example, the most popular male sterility line in hybrid rice breeding in China is II-32A, from which more than 200 hybrids have been released in recent 20 years (http://www.ricedata.cn/variety/). The results showed that out of the 18 lines, 16 have *Pid3* alleles identical to Hap_6, while the alleles of remaining two lines, Tianfeng A and V20A, were identical to Hap_21. In addition, these backbone parental lines all conferred resistance to the *M*. *oryzae* strain Zhong-10-8-14, and the transcripts of the *Pid3* alleles could be obviously detected in these lines (Supplementary Figure S3). These results demonstrated that Hap_6 of *Pid3* has been widely utilized for hybrid rice breeding in China.

Moreover, by using re-sequencing data of hybrid rice^12^, we investigated nucleotide polymorphisms of *Pid3* in 1495 hybrid rice varieties, which included 1,439 hybrid varieties from *indica-indica* crosses, 18 from *indica-japonica* crosses, and 38 from *japonica-japonica* crosses^12^. A total of 11 nucleotide polymorphism sites were identified in these hybrid rice varieties, all of which were included in the 71 sites (Supplementary Table S7). Only 88 hybrid rice varieties were found containing heterozygous sequences of *Pid3*; the remaining 1407 *Pid3* alleles belonged to four haplotypes, of which Hap_H1 was the most common haplotype shared by 1392 hybrid rice varieties. Because of low sequencing coverage (approximate 2×)^12^, it was impossible to get full sequences of *Pid3* in these 1407 hybrid rice varieties, though all variations of the Hap_H1 at the 11 nucleotide polymorphism sites were identical to Hap_6. As a result, we have reason to believe that the *Pid3* alleles of Hap_6 have prevailed in hybrid rice varieties in China.

## Discussion

So far, a great quantity of rice blast *R* genes have been identified and cloned, and almost all the cloned blast *R* genes have been applied to rice blast resistance breeding via *R* gene- self based or -tightly linked markers^48–51^. However, considering the possibility of a variety of functional alleles of the known blast *R* genes in rice populations, before a specific *R* gene is used for introgression, an accurate evaluation of its alleles in recurrent parental lines is in need. Moreover, some superior alleles, if any, could be identified by precise evaluation of the cloned *R* loci in rice germplasms^29, 46, 52^. Usually, there are three ways can be taken to evaluate a cloned *R* locus: first, certain markers which are always used in the MAS procedure for *R* loci can be applied. Then, the coding sequence fragment(s) amplified based on the cloned *R* gene should be examined. Finally, the complete *R* gene coding sequence(s) of every donor should be evaluated. However, due to the complicated and variable structure of these *NBS-LRR* type *R* genes, it is impossible to obtain accurate distribution of these cloned *R* genes just by markers and CDS fragments since single SNP could lead to the loss of function^44, 53^. Therefore, it is necessary to get the entire *R* gene sequences for their function assessment.

For the majority of rice blast R genes, it is rather difficult to obtain full sequences of alleles/orthologs by allele mining approach, due to the complexity of gene structure and vulnerable variations. However, the *Pid3* locus, as mentioned above, is a typical NBS-LRR type gene, and it is relatively uncomplicated, because it is single-copy and intronless. Moreover, our former study^29^ has revealed that alleles/orthologs of *Pid3* in other rice germplasms, even from wild rice lines, contained no InDel or structure variations (SVs), so it is practical for evaluation of *Pid3* in rice resources by exploring the existing NGS data. In this study, we analyzed nearly 3,000 *Pid3* alleles in cultivated rice accessions mainly based on the 3 K RGP sequencing data, in which each genome had an average sequencing depth of 14× with averaged genome coverage and mapping rates of 94.0% and 92.5%, respectively. Except for 16 alleles with an 8-bp insertion at the position 2461, the remaining alleles revealed no obvious InDel polymorphisms. In the coding region of the 2621 homozygous *Pid3* alleles, a total of 71 polymorphic sites were identified. By comparing sequences of *Pid3* alleles obtained from the PCR-based allele mining approach^29^, we found that most polymorphisms of *Pid3* in cultivated rice accessions were included in these 71 sites. Recently, in another study, the sequence variations of *Pid3* in 80 Yunnan rice landraces were analyzed by PCR-based allele mining approach^54^, in which a total of 39 nucleotide variations were found in the coding region of *Pid3* alleles and no InDel or SV variations were identified. By comparing the positions of nucleotide variation, we found in that study, except for 8 sites, the remaining 31 were all involved in the 71 sites. Moreover, the haplotype 8 with the highest frequency (28.8%) in that study is identical to the most common Hap_6 in our work. These results demonstrated that it was feasible to analyze the sequence variations of *Pid3* locus by utilization of the 3 K RGP sequencing data.

Some studies have shown that rice blast resistance is also correlated with the changes of some *R* gene expressions^42, 43^. In this study, our analyzing was only focused on the *Pid3* coding sequences. Because most sequence variations existed in the promoter regions, they are difficult to be further judged for their relationship with the expression changes of the corresponding *Pid3* alleles. Nevertheless, in some cultivars we checked the expression levels of *Pid3* alleles (Supplementary Figure S3) since it is possible that the loss of blast resistance in certain haplotypes might be caused by sequence variations in their promoter regions.

Of note, in this work, a total of 2953 *Pid3* alleles were tested for the premature mutation at the nucleotide position 2209. It was found that 22 (1.3%) of 1732 *indica*, 715 (83.2%) of 859 *japonica* and 40 (11%) of 362 other types carried the premature mutation. Moreover, haplotype analysis demonstrated that although Hap_2 (which was carried by 95 rice accessions and made up mostly of *japonica* lines) did not contain the premature mutation at the position 2209, it was still a pseudogene due to the premature stop codon at the position 1766. In addition, we checked the premature mutation at the position 2209 in 149 widely cultivated *japonica* varieties in north China with CAPS marker. The results showed that except for 12 accessions, all the other *japonica* cultivars carried the premature mutation. These results clearly demonstrated that *Pid3* alleles in most of these *japonica* rice cultivars were non-functional, leaving a great opportunity for utilization of functional *Pid3* alleles to improve their blast resistance. For example, the *japonica* variety Kongyu 131, the most important cultivar in north China (http://www.ricedata.cn/variety/), has not contained a functional *Pid3* allele yet (Supplementary Table S1).

Similarly, in 2621 cultivated rice lines, merely 40 haplotypes of *Pid3* were identified, and most haplotypes in group I and group II were similar to Hap_9 and Hap_6, respectively, and distinguished only by one to two SNPs. Until now, we have verified four functional *Pid3* alleles (Hap_6, Hap_13, Hap_14 and Hap_20) from cultivated rice. Of them, Hap_14 has the broadest resistance spectrum^29^. For the remaining 16 haplotypes which encode full length CC-NBS-LRR proteins, it is important to assay their resistance functionality and spectrum. We may suggest that due to merely amino acids variations, most of them probably have resistance function but the spectrum are not distinct from those verified alleles. It is noteworthy that, recently, we made a reverse mutation (T-G) at the position 2209 in Hap_9, and found that this point mutant construct had no resistance function yet. So the remaining five amino acid variations at the position 153, 204, 515, 669 and 670 are probably important to its function (Fig. 5). Moreover, in contrast to wild rice accessions where a total of 101 polymorphic sites were identified in just 17 wild lines, there were only 71 polymorphic sites detected in a total of 2621 cultivated rice lines. This contrasting picture unambiguously showed that the genetic diversity at the *Pid3* locus in cultivated rice lines has been restricted greatly. In order to explore more superior alleles at the *Pid3* locus, we must focus on wild rice accessions in future. In our previous study, three orthologs of *Pid3* with broad resistance spectrum were cloned from wild rice accessions^29^. In fact, many rice *R* genes have been identified from wild rice, including the two well-known rice *R* genes, *Xa21* for resistance to *Xanthomonas oryzae* and *Pi9* for resistance to *M*. *oryzae* originated from the wild rice *O*. *longistaminat* and *O*. *minuta* respectively^37, 55^. They both showed broader-spectrum resistances to pathogens and have been widely applied to rice breeding. Finally, considering that Hap_6, the most common haplotype of *Pid3*, has been widely deployed in Chinese *indica* rice breeding especially in the major hybrid rice cultivars^56^, here we would suggest that it is the time to introduce some novel blast resistance genes (alleles) into the rice varieties with *indica* background^57^.

## Materials and Methods

### Re-sequencing data of *Pid3* alleles

Data of SNPs and InDels at the *Pid3* locus in 3,000 rice accessions were downloaded from the Rice SNP-Seek Database^18^ (http://oryzasnp.org/iric-portal/index.zul) and the RMB database^58^ (http://www.rmbreeding.cn/snp3k). The 2775-bp coding sequence of *Pid3* corresponds to the region (13055256-13058027) on chromosome 6 of the *Nipponbare* genome in the 3,000 rice genome project sequencing data. Sequence variations at the *Pid3* locus in 1495 hybrid rice varieties were obtained from the RiceHap4 database^12^ (http://202.127.18.228/RiceHap4/index.php).

### Plant materials

A set of 289 cultivated varieties including 140 *indica* varieties and 149 *japonica* varieties (Supplementary Table S1) were selected from China for detection of the nonsense mutation of *Pid3* at the nucleotide position 2209 by the CAPS marker^44^. All of the rice varieties were kept in our lab. The susceptible recipient TP309 was used for transformation of the Hap_6 of *Pid3*. The varieties were cultivated in the experimental field of the Hunan Hybrid Rice Research Center in Changsha under normal growing conditions.

### *M. oryzae* isolates

125 *M*. *oryzae* isolates used in this study were collected from rice fields around China, and were kindly provided by Dr.Yunliang Peng of Sichuan Academy of Agricultural Sciences^59^ and by Dr. Cailin Lei of Institute of Crop Sciences, Chinese Academy of Agricultural Sciences^44^. The diagnostic isolate of *M*. *oryzae* Zhong-10-8-14 was used for the phenotypic evaluation of the backbone parental lines of hybrid rice varieties in China, and the remaining isolates were used to assay the resistance spectra of *Pid3* and Hap_6.

### Detection of the nonsense mutation by the CAPS marker

Genomic DNA were extracted from fresh leaves of the 289 rice varieties using modified CTAB method of DNA isolation. A 658-bp fragment was amplified using the primer pair (Pid3CF: 5′-TACTACTCATGGAAGCTAGTTCTC-3′ and Pid3CR: 5′-ACGTCACAAATCATTCGCTC-3′). PCR amplification was carried out using the following profile: initial DNA denaturation at 95 °C for 4 min; followed by 35 cycles of denaturation at 95 °C for 30 s, annealing at 58 °C for 30 s, and extension at 72 °C for 30 s; and final extension at 72 °C for 5 min. PCR products were digested with the restriction endoenzyme *Bam*HI. The absence of a 506-bp restriction fragment was considered to represent the nonsense mutation at the position 2209.

### Sequence analysis

Sequences were aligned using CLUSTAL X version 2.0^60^ and adjusted manually with Microsoft office excel 2010. Nucleotide diversity π (average number of nucleotide differences per site), π_non_/π_syn_ (average ratio of non-synonymous site diversity over synonymous site diversity) and haplotype diversity analysis were calculated using DNASP v5.0^61^. Haplotype flowchart was constructed with the computer program Network 5.0 (http://www.fluxus-engineering.com/sharenet.htm). DNASP v5.0 was also used to perform Tajima’s D test and sliding-window analysis of *Pid3* alleles.

### DNA sequencing

DNA was extracted from fresh leaves of the 18 *indica* and 12 *japonica* rice varieties. Primers (Pid3SF: 5′-AGTAACACCCAAGGATAGGATAG-3′ and Pid3SR: 5′-GAACGACAAGTGCGACATGATTG-3′) that amplified the full coding sequence of *Pid3* were designed according to *Pid3* sequence in rice variety Digu. PCR amplification was carried out using the following profile: initial DNA denaturation, 95 °C for 4 min; followed by 30 cycles of denaturation, 95 °C for 30 s; annealing, 58 °C for 30 s; extension, 72 °C for 3 min; and final extension at 72 °C for 5 min. The PCR products were sequenced by TsingKe Biology Technology.

### Vector construction and Rice transformation

For the Hap_6 overexpression test, primer pair (Pid3OF: 5′-TTTCTAGAAGTAACACCCAAGGATAGGATAG-3′ and Pid3OR: 5′-CTGTCGACGAACGACAAGTGCGACATGATTG-3′) were designed to amplify the coding sequence of *Pid3* allele from genomic DNA of cultivar 93-11. An *Xba*I and an *Sal*I recognition site (underlined) with two protecting bases (TT and CT) were added to their 5′ ends, respectively, then the PCR product was cloned into the binary vector pZH01 through the *Xba*I and *Sal*I cloning sites. For the Hap_6 complementation test, the 6236-bp genomic sequence of *Pid3* allele containing the promoter region and the full coding region was amplified from genomic DNA of 93-11 using the primer pair (Pid3FF: 5′-GGGTACCCCACACATTGTACACCTACGACCAC-3′ and Pid3FR: 5′-CCCCGGGGGAACGACAAGTGCGACATGATTG-3′), and then cloned into the binary vector pMNDRBBin6^62^ through the *KpnI* and XmaI cloning sites (underlined). After sequence verification the final constructs were introduced into *Agrobacterium tumefaciens* LBA4404. The callus of susceptible *japonica* variety TP309 was transformed according to published methods^63^. The resistance of the primary transgenic lines (T0) was challenged by inoculation with the *M*. *oryzae* strain Zhong-10-8-14.

### Expression analysis of *Pid3* alleles

RNA was isolated from leaf tissue with the TRIzol reagent (Invitrogen, Carlsbad, CA), and cDNA was synthesized from poly(A) + RNA using a cDNA synthesis kit (Transgen, Beijing). RT-PCR was performed with the specific primer pair Pid3C for 30 cycles of amplification. PCR amplification was as follows: 95 °C for 4 min; followed by 30 cycles of denaturation, 95 °C for 30 s; annealing, 58 °C for 30 s; extension, 72 °C for 30 s; and final extension at 72 °C for 5 min. Transcription of the *actin* gene was used to normalize the cDNA levels with the primer pair 5′-AGCAACTGGGATGATATGGA-3′ and 5′-CAGGGCGATGTAGGAAAGC-3′. Amplification of the actin gene was conducted for 27 cycles and the annealing temperature was 57 °C.

### Fungal inoculation

Six to eight plants were tested for each cultivar. Disease reaction to blast followed the modified standard pathogenicity assay as previously described^31^. Specifically, Rice seedlings at the four-leaf stage were inoculated by spraying a spore suspension (5 × 10^4^ spores/ml) of the *M.oryzae* isolates onto the leaves in a plastic bag. After inoculation plastic bags were sealed to maintain at 25 °C and 100% humidity in the dark for 24 h. Subsequently, plants were moved to the greenhouse (the humidity was maintained 70–85%, the temperature was 23/28 °C, and the lighting was 14/10 h for light/dark). and were allowed to grow to permit the development of expected disease symptoms. The disease reaction was examined one week after inoculation with the susceptible variety, TP309, as a control. The disease reaction was rated as 0–5, 0–3 as resistance and 4–5 as susceptible based on visual number and amount of lesions at the second youngest leaf ^52^.

## Electronic supplementary material

Supplementary information
