# Supplementary material for: Allelic variation of the rice blast resistance gene Pid3 in cultivated rice worldwide
Source: Sci Rep. 2017 Sep 4;7:10362. doi: 10.1038/s41598-017-10617-2 (PMC5583387; doi:10.1038/s41598-017-10617-2)
Supplement: Supplementary file 1 — Supplementary information [file 41598_2017_10617_MOESM1_ESM.pdf]

# Allelic variation of the rice blast resistance gene *Pid3* in cultivated rice worldwide

Qiming Lv[1, 2], Zhiyuan Huang[1], Xiao Xu[2], Li Tang[1], Hai Liu[1] , Chunchao Wang[3], Zhuangzhi Zhou[2], Yeyun Xin[1], Junjie Xing[1], Zhirong Peng[1], Xiaobing Li[2], Tianqing Zheng[3]*, Lihuang Zhu[1, 2]*

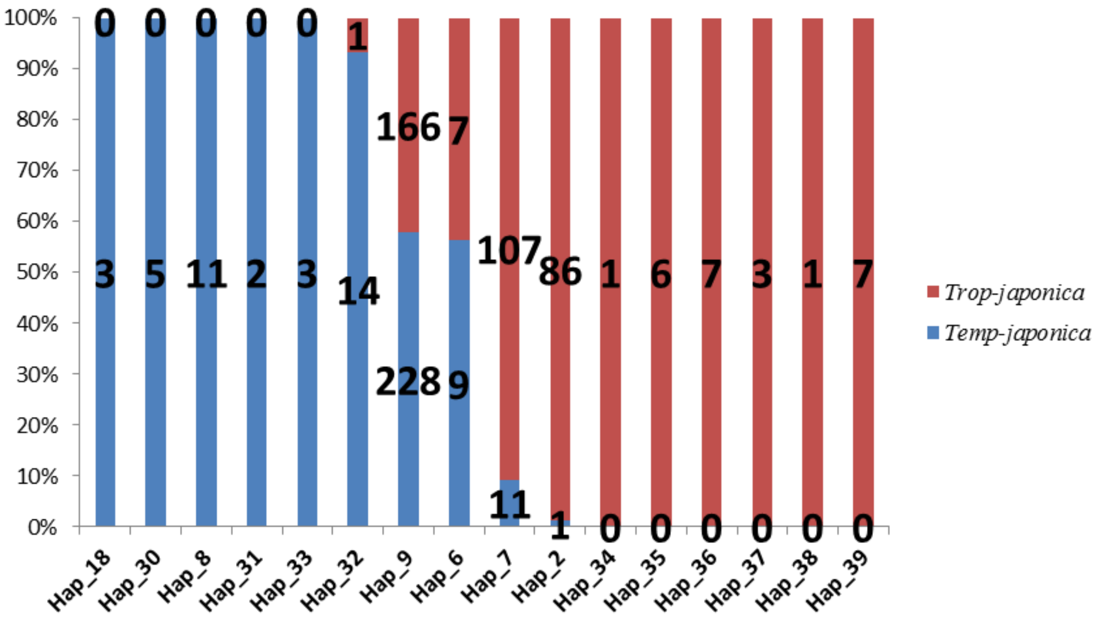


Figure S1. Haplotypes of *Pid3* in tropical and temperate *japonica* rice accessions

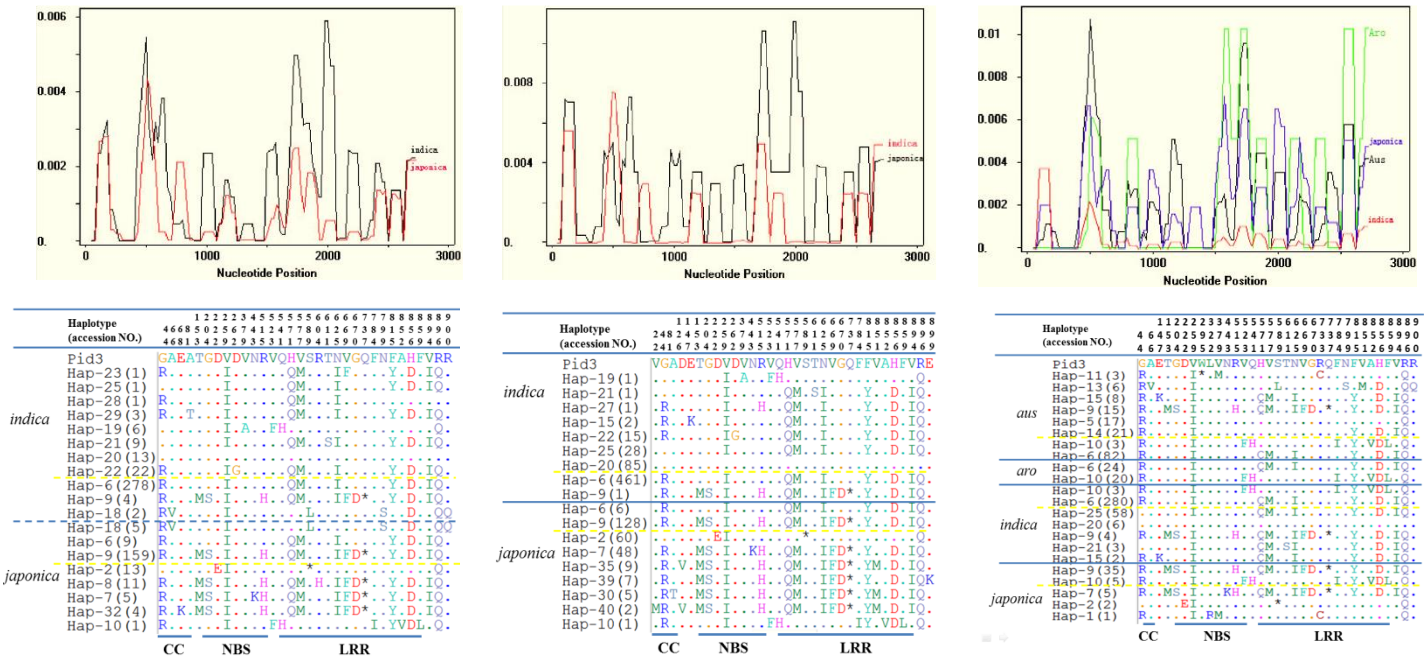


Figure S2. Nucleotide diversity and amino acid variations of *Pid3* in different regions

Left: East Asia; Middle: Southeast Asia; Right: South Asia

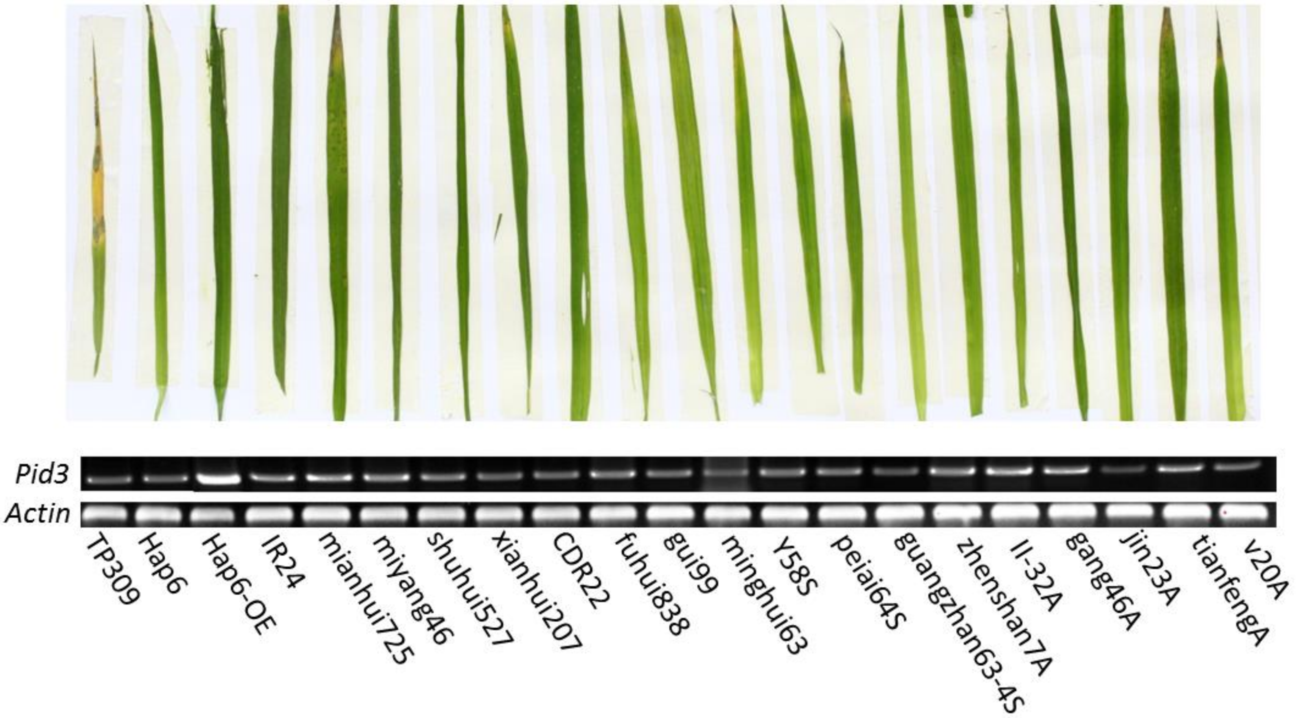


Figure S3. Backbone parent lines of hybrid rice varieties conferred resistance to *M. oryzae* zhong-10-8-14

Table S1. Detection of the premature mutation at the position 2209 by CAPS marker in 289 rice varieties.

| varieties | nucleotide at the position 2209 | subpopulation | country |
|---|---|---|---|
| P88S | G | *indica* | China |
| shen08S | G | *indica* | China |
| 1892S | G | *indica* | China |
| C815S | G | *indica* | China |
| PA64S | G | *indica* | China |
| Y58S | G | *indica* | China |
| guangzhan63S-2 | G | *indica* | China |
| zhunS | G | *indica* | China |
| zhu1S | G | *indica* | China |
| guangzhan63-4S | G | *indica* | China |
| xiangling628S | G | *indica* | China |
| 638S | G | *indica* | China |
| tianfengA | G | *indica* | China |
| shen95A | G | *indica* | China |
| II-32A | G | *indica* | China |
| T98A | G | *indica* | China |
| wufengA | G | *indica* | China |
| fengyuanA | G | *indica* | China |
| yuetaiA | G | *indica* | China |
| jin23A | G | *indica* | China |
| bing1A | G | *indica* | China |
| V20A | G | *indica* | China |
| YTB | G | *indica* | China |
| jin23B | G | *indica* | China |
| fengyuanB | G | *indica* | China |
| V20B | G | *indica* | China |
| T98B | G | *indica* | China |
| G46B | G | *indica* | China |
| II-32B | G | *indica* | China |
| zhenshan97B | G | *indica* | China |
| fuyiB | G | *indica* | China |
| D62B | G | *indica* | China |
| R0293 | G | *indica* | China |
| R084 | G | *indica* | China |
| R111 | G | *indica* | China |
| R128 | G | *indica* | China |
| R158 | G | *indica* | China |
| R163 | G | *indica* | China |
| R183 | G | *indica* | China |
| R197 | G | *indica* | China |
| R227 | G | *indica* | China |

| | | | |
|---|---|---|---|
| R265 | G | *indica* | China |
| R288 | G | *indica* | China |
| R299 | G | *indica* | China |
| shuhui498 | G | *indica* | China |
| shuhui527 | G | *indica* | China |
| R644 | G | *indica* | China |
| mianhui725 | G | *indica* | China |
| R8006 | G | *indica* | China |
| R838 | G | *indica* | China |
| R86 | G | *indica* | China |
| R88 | G | *indica* | China |
| R5550 | G | *indica* | China |
| 93-11 | G | *indica* | China |
| gui99 | G | *indica* | China |
| jusuidao | G | *indica* | China |
| miyang46 | G | *indica* | China |
| minghui63 | G | *indica* | China |
| xianhui207 | G | *indica* | China |
| yuanhui2hao | G | *indica* | China |
| nongxiang16 | G | *indica* | China |
| nongxiang18 | G | *indica* | China |
| nongxiang24 | G | *indica* | China |
| nongxiang26 | G | *indica* | China |
| nongxiang28 | G | *indica* | China |
| nongxiang29 | G | *indica* | China |
| huanghuazhan | G | *indica* | China |
| huangguangzhan | G | *indica* | China |
| xiangyaxiangzhan | G | *indica* | China |
| yuetaisimiao | G | *indica* | China |
| xiangwan12 | G | *indica* | China |
| xiangwan13 | G | *indica* | China |
| xiangwan17 | G | *indica* | China |
| xing1hao | G | *indica* | China |
| xing2hao | G | *indica* | China |
| yuzhenxiang | G | *indica* | China |
| mianhui662 | G | *indica* | China |
| mianhui3724 | G | *indica* | China |
| neihui99-14 | G | *indica* | China |
| mianhui146 | G | *indica* | China |
| mianhui523 | G | *indica* | China |
| mianhui9939 | G | *indica* | China |
| mianhui3728 | G | *indica* | China |
| luhui602 | G | *indica* | China |

| | | | |
|---|---|---|---|
| chuanR527 | G | *indica* | China |
| chuanR838 | G | *indica* | China |
| CDR22 | G | *indica* | China |
| shuhui166 | G | *indica* | China |
| shuihui118 | G | *indica* | China |
| chenghui727 | G | *indica* | China |
| chuanhui687 | G | *indica* | China |
| chuanR240 | G | *indica* | China |
| xiangR1128 | G | *indica* | China |
| R907 | G | *indica* | China |
| R702 | G | *indica* | China |
| zhehui0506 | G | *indica* | China |
| R507 | G | *indica* | China |
| R925 | G | *indica* | China |
| R516 | G | *indica* | China |
| R814 | G | *indica* | China |
| T136 | G | *indica* | China |
| R336 | G | *indica* | China |
| R332 | G | *indica* | China |
| R542 | G | *indica* | China |
| R6547 | G | *indica* | China |
| R153 | G | *indica* | China |
| R902 | G | *indica* | China |
| mingtianxianhui | G | *indica* | China |
| R2012 | G | *indica* | China |
| R28 | G | *indica* | China |
| R9368 | G | *indica* | China |
| luobiao | G | *indica* | China |
| zhongjiangD208 | G | *indica* | China |
| yangdao4hao | G | *indica* | China |
| yangdao8hao | G | *indica* | China |
| xiangxian164 | G | *indica* | China |
| R207 | G | *indica* | China |
| fenghuazhan | G | *indica* | China |
| huazhan | G | *indica* | China |
| guangchao924 | G | *indica* | China |
| yuekang0924 | G | *indica* | China |
| yuekang1124 | G | *indica* | China |
| guangchao123 | G | *indica* | China |
| R6090 | G | *indica* | China |
| yueR9113 | G | *indica* | China |
| xin1122 | G | *indica* | China |
| wushansimiao | G | *indica* | China |

| | | | |
|---|---|---|---|
| IR64 | G | *indica* | China |
| R6326 | G | *indica* | China |
| xianghui4hao | G | *indica* | China |
| xianghui1hao | G | *indica* | China |
| IR71033 | G | *indica* | China |
| yangdao1hao | G | *indica* | China |
| yangdao2hao | G | *indica* | China |
| BG902 | G | *indica* | China |
| yan3021 | G | *indica* | China |
| zhongjian2hao | G | *indica* | China |
| duoxi1hao | G | *indica* | China |
| IRBB61 | G | *indica* | China |
| wenhui689 | G | *indica* | China |
| lianjing6hao | A | *japonica* | China |
| lianjing7hao | A | *japonica* | China |
| lianjing9hao | A | *japonica* | China |
| lianjing10hao | A | *japonica* | China |
| lianjing11hao | A | *japonica* | China |
| huaidao5hao | A | *japonica* | China |
| huaidao11hao | A | *japonica* | China |
| huainuo12hao | A | *japonica* | China |
| huaidao14hao | A | *japonica* | China |
| huaidao15hao | A | *japonica* | China |
| yanjing11hao | A | *japonica* | China |
| yandao815 | A | *japonica* | China |
| yangjing805 | A | *japonica* | China |
| xiangjing49 | A | *japonica* | China |
| yangjing4038 | A | *japonica* | China |
| yangjing4227 | A | *japonica* | China |
| yangjing806 | A | *japonica* | China |
| zhendao88 | A | *japonica* | China |
| zhendao9424 | A | *japonica* | China |
| ning9108 | A | *japonica* | China |
| ning5055 | A | *japonica* | China |
| nanjing46 | A | *japonica* | China |
| wuyunjing8hao | A | *japonica* | China |
| wuyunjing21hao | A | *japonica* | China |
| wuyunjing23hao | A | *japonica* | China |
| wuyunjing24hao | A | *japonica* | China |
| sidao785 | A | *japonica* | China |
| huajing5hao | A | *japonica* | China |
| huajing6hao | A | *japonica* | China |
| shengdao15 | A | *japonica* | China |

| | | | |
|---|---|---|---|
| shengdao16 | A | *japonica* | China |
| yangguang600 | A | *japonica* | China |
| lindao16 | A | *japonica* | China |
| lindao17 | A | *japonica* | China |
| xindao20 | A | *japonica* | China |
| xindao29 | A | *japonica* | China |
| jindao1007 | A | *japonica* | China |
| jingdao263 | A | *japonica* | China |
| suxiu10 | A | *japonica* | China |
| jia02147 | A | *japonica* | China |
| yongzhi19 | A | *japonica* | China |
| C418 | A | *japonica* | China |
| zhongchao123 | A | *japonica* | China |
| jijing83 | A | *japonica* | China |
| changbai16 | A | *japonica* | China |
| longjing05-191 | A | *japonica* | China |
| longjing25 | A | *japonica* | China |
| longjing26 | A | *japonica* | China |
| liaojing21 | A | *japonica* | China |
| ningjing38 | A | *japonica* | China |
| ningjing43 | A | *japonica* | China |
| danjing06-8 | A | *japonica* | China |
| jia09-90 | A | *japonica* | China |
| changshu6-85 | A | *japonica* | China |
| ningjing24 | A | *japonica* | China |
| liaoyuan31-1 | A | *japonica* | China |
| liaoyuan31-2 | A | *japonica* | China |
| jijing80 | A | *japonica* | China |
| jindao253 | A | *japonica* | China |
| tianjing28-1 | A | *japonica* | China |
| taiwan30 | A | *japonica* | China |
| taiwan50 | A | *japonica* | China |
| taiwan65 | A | *japonica* | China |
| tiejing7hao | A | *japonica* | China |
| yujing6hao | A | *japonica* | China |
| jiahe218 | A | *japonica* | China |
| kendao11 | A | *japonica* | China |
| kendao12 | A | *japonica* | China |
| kendao13 | A | *japonica* | China |
| kendao16 | A | *japonica* | China |
| kendao20 | A | *japonica* | China |
| kenjiandao6hao | A | *japonica* | China |
| kenjiandao13hao | A | *japonica* | China |

| | | | |
|---|---|---|---|
| kenjiandao14hao | A | *japonica* | China |
| kongyu131 | A | *japonica* | China |
| kennuo1hao | A | *japonica* | China |
| kennuo2hao | A | *japonica* | China |
| longjing8hao | A | *japonica* | China |
| longjing20hao | A | *japonica* | China |
| longjing21hao | A | *japonica* | China |
| longjing24hao | A | *japonica* | China |
| longjing25hao | A | *japonica* | China |
| longjing26hao | A | *japonica* | China |
| longjing27hao | A | *japonica* | China |
| longjing28hao | A | *japonica* | China |
| hejing1hao | A | *japonica* | China |
| jinxuan1hao | A | *japonica* | China |
| beidao4hao | A | *japonica* | China |
| beidao5hao | A | *japonica* | China |
| suijing4hao | A | *japonica* | China |
| suijing9hao | A | *japonica* | China |
| suijing13hao | A | *japonica* | China |
| longnuo2hao | A | *japonica* | China |
| longjingxiang1hao | A | *japonica* | China |
| longqingdao1hao | A | *japonica* | China |
| puyou18 | A | *japonica* | China |
| puyou52 | A | *japonica* | China |
| puyoudaohuaxiang2hao | A | *japonica* | China |
| puxuan28 | A | *japonica* | China |
| heizhenzhu1hao | A | *japonica* | China |
| shangyu397 | A | *japonica* | China |
| wuyoudao1hao | A | *japonica* | China |
| daohuaxiangzao2hao | A | *japonica* | China |
| songjing3hao | A | *japonica* | China |
| songjing5hao | A | *japonica* | China |
| songjing6hao | A | *japonica* | China |
| songjing7hao | A | *japonica* | China |
| songjing9hao | A | *japonica* | China |
| songjing10hao | A | *japonica* | China |
| songjing12hao | A | *japonica* | China |
| song5120 | A | *japonica* | China |
| dongnong415 | A | *japonica* | China |
| dongnong418 | A | *japonica* | China |
| dongnong419 | A | *japonica* | China |
| dongnong423 | A | *japonica* | China |
| dongnong425 | A | *japonica* | China |

| | | | |
|---|---|---|---|
| dongnong426 | A | *japonica* | China |
| dongnong427 | A | *japonica* | China |
| dongnong428 | A | *japonica* | China |
| dongnong429 | A | *japonica* | China |
| dongnong430 | A | *japonica* | China |
| tongyu207 | A | *japonica* | China |
| nongda18 | A | *japonica* | China |
| tongyuan6hao | A | *japonica* | China |
| mudanjing30 | A | *japonica* | China |
| liaojing371 | A | *japonica* | China |
| changbai12 | A | *japonica* | China |
| mudanjiang29 | A | *japonica* | China |
| jiudao63 | A | *japonica* | China |
| jiudao59 | A | *japonica* | China |
| nongda7hao | A | *japonica* | China |
| tongyu211 | A | *japonica* | China |
| jijing505 | A | *japonica* | China |
| mudanjiang31 | A | *japonica* | China |
| longdao5hao | A | *japonica* | China |
| longdao7hao | A | *japonica* | China |
| longdao8hao | A | *japonica* | China |
| nanjing49 | G | *japonica* | China |
| wuyunjing27hao | G | *japonica* | China |
| jijing88 | G | *japonica* | China |
| ningjing28 | G | *japonica* | China |
| ningjing35 | G | *japonica* | China |
| ningjing37 | G | *japonica* | China |
| ningjing41 | G | *japonica* | China |
| jinyuan45 | G | *japonica* | China |
| wuyoudao4hao | G | *japonica* | China |
| jite639 | G | *japonica* | China |
| jijing86 | G | *japonica* | China |
| mudanjiang20 | G | *japonica* | China |

Table S2. Cultivated rice accessions with 8-bp insertion at 13055566 on Chr.6

| JAPONICA NIPPONBARE POSITIONS | IRIS ID | SUBPOPULATION | 1305556 | | | | | | | |
|---|---|---|---|---|---|---|---|---|---|---|
| CEA 3::IRGC 116965-1 | IRIS 313-10260 | indx | A | T | A | T | A | T | T | C |
| ZH5 | CX305 | ind1B | A | T | A | T | A | T | T | C |
| CT 9737-6-1-1-2-2P-M::IRGC 117330-1 | IRIS 313-10352 | indx | A | T | A | T | A | T | T | C |
| YA NONG ZAO 4::IRGC 63908-1 | IRIS 313-10171 | ind1A | A | T | A | T | A | T | T | C |
| IRGA 959-1-2-2F-4-1-4A-6-CA-6X::IRGC 117006-1 | IRIS 313-10301 | indx | A | T | A | T | A | T | T | C |
| LIU TIAO XIAN::IRGC 72758-1 | IRIS 313-11881 | ind1A | A | T | A | T | A | T | T | C |
| DENG DENG QI::IRGC 72036-1 | IRIS 313-10190 | ind1A | A | T | A | T | A | T | T | C |
| YOUNIAN | B224 | ind1A | A | T | A | T | A | T | T | C |
| EPEAL 102::IRGC 78698-1 | IRIS 313-10220 | indx | A | T | A | T | A | T | T | C |
| CICA 8::C1 | IRIS 313-7664 | ind1B | A | T | A | T | A | T | T | C |
| IA CUBA 17::IRGC 116990-1 | IRIS 313-10271 | indx | A | T | A | T | A | T | T | C |
| BAI RI XIAN::IRGC 72588-1 | IRIS 313-11878 | ind1A | A | T | A | T | A | T | T | C |
| AMERICAN RICE | B035 | indx | A | T | A | T | A | T | T | C |
| SEBERANG | CX147 | indx | -/A | -/T | -/A | -/T | -/A | -/T | -/T | -/C |
| LIUSHIZAO | B163 | ind1A | -/A | -/T | -/A | -/T | -/A | -/T | -/T | -/C |
| 571::IRGC 48493-1 | IRIS 313-8872 | japx | -/A | -/T | -/A | -/T | -/A | -/T | -/T | -/C |

Table S3. Summary of synonymous and non-synonymous sites, rate of non-synonymous substitution by synonymous substitution with reference to *Pid3-w5*

| **Haplotype** | **Full Coding Region** | | | **CC Domain** | | | **NBS Domain** | | | **LRR Domain** | | |
|---|---|---|---|---|---|---|---|---|---|---|---|---|
| | SynDif | NSynDif | Ka/Ks | SynDif | NSynDif | Ka/Ks | SynDif | NSynDif | Ka/Ks | SynDif | NSynDif | Ka/Ks |
| Hap_6 | 4 | 10 | 0.75 | 0 | 1 | - | 3 | 0 | 0.00 | 0 | 7 | - |
| Hap_9 | 4 | 16 | 1.19 | 0 | 1 | - | 4 | 2 | 0.14 | 0 | 10 | - |
| Hap_7 | 4 | 17 | 1.27 | 0 | 1 | - | 4 | 3 | 0.20 | 0 | 10 | - |
| Hap_20 | 5 | 9 | 0.54 | 1 | 2 | 0.65 | 4 | 1 | 0.07 | 0 | 4 | - |
| Hap_25 | 4 | 11 | 0.83 | 0 | 2 | - | 3 | 0 | 0.00 | 0 | 7 | - |
| Hap_2 | 6 | 8 | 0.40 | 1 | 2 | 0.65 | 4 | 1 | 0.07 | 1 | 4 | 1.25 |
| Hap_22 | 4 | 11 | 0.83 | 0 | 1 | - | 3 | 1 | 0.09 | 0 | 7 | - |
| Hap_10 | 6 | 11 | 0.55 | 0 | 1 | - | 3 | 0 | 0.00 | 1 | 8 | 2.53 |
| Hap_14 | 5 | 7 | 0.42 | 0 | 1 | - | 4 | 0 | 0.00 | 0 | 4 | - |
| Hap_5 | 6 | 6 | 0.30 | 0 | 1 | - | 4 | 0 | 0.00 | 1 | 4 | 1.25 |
| Hap_21 | 4 | 12 | 0.89 | 0 | 2 | - | 3 | 0 | 0.00 | 0 | 8 | - |
| Hap_8 | 4 | 17 | 1.27 | 0 | 1 | - | 4 | 2 | 0.14 | 0 | 11 | - |
| Hap_19 | 4 | 10 | 0.75 | 1 | 2 | 0.65 | 3 | 1 | 0.09 | 0 | 5 | - |
| Hap_32 | 4 | 17 | 1.27 | 0 | 2 | - | 4 | 2 | 0.14 | 0 | 10 | - |
| Hap_15 | 4 | 11 | 0.83 | 0 | 1 | - | 3 | 0 | 0.00 | 0 | 7 | - |
| Hap_36 | 4 | 18 | 1.35 | 0 | 1 | - | 4 | 4 | 0.27 | 0 | 10 | - |
| Hap_35 | 5 | 16 | 0.95 | 0 | 1 | - | 5 | 2 | 0.11 | 0 | 9 | - |
| Hap_18 | 1 | 1 | 0.31 | 0 | 0 | - | 0 | 0 | - | 1 | 1 | 0.33 |
| Hap_39 | 4 | 17 | 1.27 | 0 | 1 | - | 4 | 2 | 0.14 | 0 | 10 | - |
| Hap_11 | 6 | 9 | 0.45 | 1 | 1 | 0.32 | 5 | 2 | 0.11 | 0 | 5 | - |
| Hap_30 | 4 | 16 | 1.19 | 0 | 2 | - | 4 | 2 | 0.14 | 0 | 9 | - |
| Hap_24 | 5 | 16 | 0.95 | 0 | 1 | - | 4 | 2 | 0.14 | 1 | 10 | 3.15 |
| Hap_26 | 4 | 11 | 0.83 | 0 | 1 | - | 3 | 1 | 0.09 | 0 | 7 | - |
| Hap_1 | 6 | 9 | 0.45 | 1 | 1 | 0.32 | 5 | 2 | 0.11 | 0 | 5 | - |
| Hap_29 | 4 | 11 | 0.83 | 0 | 2 | - | 3 | 0 | 0.00 | 0 | 7 | - |
| Hap_33 | 5 | 16 | 0.95 | 0 | 1 | - | 5 | 2 | 0.11 | 0 | 10 | - |
| Hap_37 | 4 | 18 | 1.35 | 0 | 1 | - | 4 | 3 | 0.20 | 0 | 11 | - |
| Hap_17 | 3 | 10 | 1.00 | 0 | 1 | - | 2 | 0 | 0.00 | 0 | 7 | - |
| Hap_31 | 5 | 16 | 0.95 | 0 | 1 | - | 4 | 2 | 0.14 | 0 | 10 | - |
| Hap_40 | 5 | 17 | 1.01 | 0 | 2 | - | 5 | 2 | 0.11 | 0 | 9 | - |
| Hap_3 | 9 | 8 | 0.27 | 0 | 1 | - | 5 | 0 | 0.00 | 3 | 6 | 0.62 |
| Hap_4 | 9 | 7 | 0.23 | 0 | 1 | - | 5 | 0 | 0.00 | 3 | 5 | 0.52 |
| Hap_12 | 4 | 10 | 0.75 | 0 | 1 | - | 3 | 0 | 0.00 | 0 | 7 | - |
| Hap_16 | 4 | 10 | 0.75 | 0 | 1 | - | 3 | 0 | 0.00 | 0 | 7 | - |
| Hap_23 | 4 | 11 | 0.83 | 0 | 1 | - | 3 | 0 | 0.00 | 0 | 8 | - |
| Hap_27 | 4 | 11 | 0.83 | 0 | 1 | - | 3 | 1 | 0.09 | 0 | 7 | - |
| Hap_28 | 4 | 10 | 0.75 | 0 | 1 | - | 3 | 0 | 0.00 | 0 | 7 | - |
| Hap_34 | 4 | 10 | 0.75 | 1 | 1 | 0.32 | 3 | 0 | 0.00 | 0 | 7 | - |
| Hap_38 | 5 | 16 | 0.95 | 0 | 1 | - | 5 | 2 | 0.11 | 0 | 10 | - |

Table S4. Nucleotide polymorphisms of *Pid3* haplotypes among cultivated and wild rice accessions

| Nucleotide position | w11-1/J1/J2/Hap9 | w11-2 | w1-1 | w1-2 | w15/Hap18 | w2 | w13 | w6 | w12/I2/Hap6 | w7 | w17 | w16 | w14 | w5/Hap13 | w4 | w3 | w9 | w10 | w8 | w13-2 | I5 | I3/Hap20 | I1/Hap14 | I4/Hap21 | J3/J5/Hap8 | J4 | Hap7 | Hap25 | Hap2 | Hap22 | Hap10 | Hap5 | Hap19 | Hap32 | Hap15 | Hap36 | Hap35 | Hap39 | Hap11 | Hap30 | Hap24 | Hap26 | Hap1 | Hap29 | Hap33 | Hap37 | Hap17 | Hap31 | Hap40 | Hap3 | Hap4 | Hap12 | Hap16 | Hap23 | Hap27 | Hap28 | Hap34 | Hap38 |
|---|---|---|---|---|---|---|---|---|---|---|---|---|---|---|---|---|---|---|---|---|---|---|---|---|---|---|---|---|---|---|---|---|---|---|---|---|---|---|---|---|---|---|---|---|---|---|---|---|---|---|---|---|---|---|---|---|---|---|
| 1 | A | A | A | G | A | A | A | A | A | A | A | G | A | A | A | A | A | A | A | A | A | A | A | A | A | A | A | A | A | A | A | A | A | A | A | A | A | A | A | A | A | A | A | A | A | A | A | A | A | A | A | A | A | A | A | A | A | A |
| 2 | T | T | T | C | T | T | T | T | T | T | T | C | T | T | T | T | T | T | T | T | T | T | T | T | T | T | T | T | T | T | T | T | T | T | T | T | T | T | T | T | T | T | T | T | T | T | T | T | T | T | T | T | T | T | T | T | T | T |
| 46 | G | G | G | G | G | G | G | G | G | G | G | G | G | G | G | G | G | G | G | G | G | G | G | G | G | A | G | G | G | G | G | G | G | G | G | G | G | G | G | G | G | G | G | G | G | G | G | G | G | G | G | G | G | G | G | G | G | G |
| 64 | G | G | G | G | G | G | G | G | G | G | G | G | G | G | G | G | G | G | G | G | G | G | G | G | G | G | G | G | G | G | G | G | G | G | G | G | G | G | G | G | G | G | G | G | G | G | G | G | A | G | G | G | G | G | G | G | G | G |
| 65 | T | T | T | T | T | T | T | T | T | T | T | T | T | T | T | C | T | T | T | T | T | T | T | T | T | T | T | T | T | T | T | T | T | T | T | T | T | T | T | T | T | T | T | T | T | T | T | T | T | T | T | T | T | T | T | T | T | T |
| 130 | C | C | C | C | C | C | C | C | C | C | C | C | C | C | C | C | C | C | C | C | C | G | C | G | C | C | C | G | G | C | C | C | G | C | C | C | C | C | C | C | C | C | C | C | C | C | C | C | C | C | C | C | C | C | C | C | C | C |
| 144 | G | G | G | T | G | G | G | T | G | T | G | T | G | G | G | G | G | T | G | G | G | T | G | G | G | G | G | G | T | G | G | G | T | G | G | G | G | G | T | G | G | G | T | G | G | G | G | G | G | G | G | G | G | G | G | G | T | G |
| 197 | C | C | C | C | T | C | C | C | C | C | C | C | C | T | C | C | C | C | C | C | C | C | C | C | C | C | C | C | C | C | C | C | C | C | C | C | C | C | C | C | C | C | C | C | C | C | C | C | C | C | C | C | C | C | C | C | C | C |
| 202 | G | G | G | G | G | G | G | G | G | G | G | G | G | G | G | G | G | G | G | G | G | G | G | G | G | G | G | G | G | G | G | G | G | A | G | G | G | G | G | G | G | G | G | G | G | G | G | G | G | G | G | G | G | G | G | G | G | G |
| 213 | C | C | C | C | C | T | C | C | C | C | C | C | C | C | C | C | C | C | C | C | C | C | C | C | C | C | C | C | C | C | C | C | C | C | C | C | C | C | C | C | C | C | C | C | C | C | C | C | C | C | C | C | C | C | C | C | C | C |
| 221 | T | T | T | T | T | T | T | T | T | C | T | T | T | T | T | T | T | T | T | T | T | T | T | T | T | T | T | T | T | T | T | T | T | T | T | T | T | T | T | T | T | T | T | T | T | T | T | T | T | T | T | T | T | T | T | T | T | T |
| 235 | A | A | A | A | A | A | A | T | A | A | A | A | A | A | A | A | A | A | A | A | A | A | A | A | A | A | A | A | A | A | A | A | A | A | A | A | A | A | A | A | A | A | A | A | A | A | A | A | A | A | A | A | A | A | A | A | A | A |
| 236 | G | T | T | G | G | G | G | G | G | G | G | G | G | G | G | G | G | G | G | G | G | G | G | G | G | G | G | G | G | G | G | G | G | G | G | G | G | G | G | G | G | G | G | G | G | G | G | G | G | G | G | G | G | G | G | G | G | G |
| 241 | G | G | G | G | G | G | G | G | G | G | G | G | G | G | G | G | G | G | G | G | G | G | G | G | G | G | G | G | G | G | G | G | G | G | G | G | G | G | G | A | G | G | G | A | G | G | G | G | G | G | G | G | G | G | G | G | G | G |
| 264 | C | C | C | C | C | C | C | C | C | C | C | C | C | C | C | C | C | T | C | C | C | C | C | C | C | C | C | C | C | C | C | C | C | C | C | C | C | C | C | C | C | C | C | C | C | C | C | C | C | C | C | C | C | C | C | C | C | C |
| 301 | C | C | C | C | C | C | C | C | C | C | C | C | T | C | C | C | C | C | C | C | C | C | C | C | C | C | C | C | C | C | C | C | C | C | C | C | C | C | C | C | C | C | C | C | C | C | C | C | C | C | C | C | C | C | C | C | C | C |

| | | | | | | | | | | | | | | | | | | | | | | | | | | | | | | | | | | | | | | | | | | | | | | | | | | | | | | | | | | |
|---|---|---|---|---|---|---|---|---|---|---|---|---|---|---|---|---|---|---|---|---|---|---|---|---|---|---|---|---|---|---|---|---|---|---|---|---|---|---|---|---|---|---|---|---|---|---|---|---|---|---|---|---|---|---|---|---|---|---|
| 303 | G | G | G | G | G | C | G | G | G | G | G | G | G | G | G | G | G | G | G | C | G | G | G | G | G | G | G | G | G | G | G | G | G | G | G | G | G | G | G | G | G | G | G | G | G | G | G | G | G | G | G | G | G | G | G | G | G | G |
| 340 | A | A | A | A | A | A | A | A | A | A | A | A | A | A | A | A | G | A | A | A | A | A | A | A | A | A | A | A | A | A | A | A | A | A | A | A | A | A | A | A | A | A | A | A | A | A | A | A | A | A | A | A | A | A | A | A | A | A |
| 377 | A | A | A | A | A | A | A | A | A | A | A | A | A | A | A | A | A | A | A | A | A | A | A | A | A | A | A | A | A | A | A | A | A | A | A | A | T | A | A | A | A | A | A | A | A | A | A | A | T | A | A | A | A | A | A | A | A | A |
| 431 | A | A | A | A | A | A | A | A | A | A | A | A | G | A | A | A | A | A | A | G | A | A | A | A | A | A | A | A | A | A | A | A | A | A | A | A | A | A | A | A | A | A | A | A | A | A | A | A | A | A | A | A | A | A | A | A | A | A |
| 435 | C | C | C | C | C | C | C | T | C | C | C | C | C | C | C | C | C | C | C | C | C | C | C | C | C | C | C | C | C | C | C | C | C | C | C | C | C | C | C | C | C | C | C | C | C | C | C | C | C | C | C | C | C | C | C | C | C | C |
| 439 | G | G | G | G | G | G | G | G | G | G | G | G | G | G | G | G | G | G | G | G | G | G | G | G | G | G | G | G | G | G | G | G | G | G | A | G | G | G | G | G | G | G | G | G | G | G | G | G | G | G | G | G | G | G | G | G | G | G |
| 447 | C | C | C | C | C | A | C | C | C | C | C | C | C | C | C | C | C | C | C | C | C | C | C | C | C | C | C | C | C | C | C | C | C | C | C | C | C | C | C | C | C | C | C | C | C | C | C | C | C | C | C | C | C | C | C | C | C | C |
| 449 | C | C | C | C | C | C | C | C | C | T | C | C | C | C | C | C | C | C | C | C | C | C | C | C | C | C | C | C | C | C | C | C | C | C | C | C | C | C | C | C | C | C | C | C | C | C | C | C | C | C | C | C | C | C | C | C | C | C |
| 453 | A | A | A | A | A | A | A | A | A | A | A | G | A | A | A | A | A | A | A | A | A | A | A | A | A | A | A | A | A | A | A | A | A | A | A | A | A | A | A | A | A | A | A | A | A | A | A | A | A | A | A | A | A | A | A | A | A | A |
| 458 | T | C | C | C | C | C | C | T | C | C | T | C | C | C | C | C | C | T | C | C | C | C | C | C | T | T | T | C | C | C | C | C | C | T | C | T | T | T | C | T | T | C | C | C | T | T | C | T | T | C | C | C | C | C | C | C | C | T |
| 477 | A | G | G | A | A | G | A | A | G | A | A | A | A | A | G | G | G | A | G | A | G | A | G | G | A | A | A | G | A | G | G | G | A | A | G | A | A | A | A | A | A | G | A | G | A | A | G | A | A | A | A | A | G | G | G | G | A | A |
| 502 | T | T | T | T | T | T | T | T | T | T | T | T | T | T | T | T | T | T | T | T | T | T | T | T | T | T | T | T | T | T | T | T | T | T | T | T | T | T | T | T | T | C | T | T | T | T | T | T | T | T | T | T | T | T | T | T | T | T |
| 525 | T | C | C | T | C | C | T | T | C | T | T | T | T | C | C | C | C | T | C | T | C | T | C | C | T | T | T | C | T | C | C | C | T | T | C | T | T | T | T | T | T | C | T | C | T | T | C | T | T | T | T | T | C | C | C | C | T | T |
| 537 | G | G | G | G | G | G | G | G | A | G | G | G | G | G | A | G | A | G | G | G | A | G | A | A | G | G | G | A | G | A | G | G | G | G | A | G | G | G | G | G | G | A | G | A | G | G | G | G | G | G | G | G | G | A | A | A | G | G |
| 572 | A | A | A | A | A | A | A | A | A | A | A | A | A | A | A | T | A | A | A | A | A | A | A | A | A | A | A | A | A | A | A | A | A | A | A | A | A | A | A | A | A | A | A | A | A | A | A | A | A | A | A | A | A | A | A | A | A | A |
| 607 | A | A | A | A | A | A | A | A | A | A | A | A | A | A | A | A | A | A | G | A | A | A | A | A | A | A | A | A | A | A | A | A | A | A | A | A | A | A | A | A | A | A | A | A | A | A | A | A | A | A | A | A | A | A | A | A | A | A |
| 610 | A | G | G | G | G | G | G | G | G | G | A | T | G | G | G | G | G | G | G | G | G | G | G | G | A | A | A | G | G | G | G | G | G | A | G | A | A | A | G | A | A | G | G | G | A | A | G | A | A | G | G | G | G | G | G | G | G | A |
| 627 | G | G | G | G | G | G | G | G | G | G | G | G | G | G | G | G | G | G | G | G | G | G | G | G | G | G | G | G | G | G | G | G | G | G | G | G | G | G | G | G | G | G | G | G | A | G | G | G | G | G | G | G | G | G | G | G | G | G |
| 641 | A | A | A | A | A | A | A | A | A | A | A | A | C | A | A | A | A | A | A | C | A | A | A | A | A | A | A | A | A | A | A | A | A | A | A | A | A | A | A | A | A | A | A | A | A | A | A | A | A | A | A | A | A | A | A | A | A | A |
| 648 | C | C | C | T | C | C | C | C | C | C | C | T | C | C | C | C | C | C | C | C | C | C | C | C | C | C | C | C | C | C | C | C | C | C | C | C | C | C | C | C | C | C | C | C | C | C | C | C | C | C | C | C | C | C | C | C | C | C |
| 666 | C | C | C | C | C | C | C | C | C | C | C | C | C | C | C | C | C | C | C | C | C | C | C | C | C | C | C | C | A | C | C | C | C | C | C | C | C | C | C | C | C | C | C | C | C | C | C | C | C | C | C | C | C | C | C | C | C | C |
| 690 | T | T | T | T | T | T | T | T | T | T | T | T | T | T | T | T | T | C | T | T | T | T | T | T | T | T | T | T | T | T | T | T | T | T | T | T | T | T | T | T | T | T | T | T | T | T | T | T | T | T | T | T | T | T | T | T | T | T |
| 705 | C | C | C | C | C | C | C | C | C | C | C | C | C | C | C | C | T | C | C | C | C | C | C | C | C | C | C | C | C | C | C | C | C | C | C | C | C | C | C | C | C | C | C | C | C | C | C | C | C | C | C | C | C | C | C | C | C | C |
| 714 | C | C | C | C | C | C | T | C | C | C | C | C | C | C | C | C | C | C | C | C | C | C | C | C | C | C | C | C | C | C | C | C | C | C | C | C | C | C | C | C | C | C | C | C | C | C | C | C | C | C | C | C | C | C | C | C | C | C |
| 725 | G | C | G | G | G | G | G | G | G | G | G | G | G | G | G | G | G | G | G | G | G | G | G | G | G | G | G | G | G | G | G | G | G | G | G | G | G | G | G | G | G | G | G | G | G | G | G | G | G | G | G | G | G | G | G | G | G | G |

| | | | | | | | | | | | | | | | | | | | | | | | | | | | | | | | | | | | | | | | | | | | | | | | | | | | | | | | | | | | |
|---|---|---|---|---|---|---|---|---|---|---|---|---|---|---|---|---|---|---|---|---|---|---|---|---|---|---|---|---|---|---|---|---|---|---|---|---|---|---|---|---|---|---|---|---|---|---|---|---|---|---|---|---|---|---|---|---|---|---|
| 731 | C | T | T | C | C | C | C | C | C | C | C | C | C | C | C | C | C | C | C | C | C | C | C | C | C | C | C | C | C | C | C | C | C | C | C | C | C | C | C | C | C | C | C | C | C | C | C | C | C | C | C | C | C | C | C | C | C | C |
| 738 | A | A | A | A | A | A | G | A | A | A | A | A | A | A | A | A | A | A | A | A | A | A | A | A | A | A | A | A | A | A | A | A | A | A | A | A | A | A | A | A | A | A | A | A | A | A | A | A | A | A | A | A | A | A | A | A | A | A |
| 756 | T | T | T | T | C | T | C | T | T | T | T | T | C | C | T | T | T | T | T | C | T | T | T | T | T | T | T | T | T | T | T | T | T | T | T | T | T | T | T | T | T | T | T | T | T | T | T | T | T | T | T | T | T | T | T | T | T | T |
| 775 | A | A | A | A | A | A | A | A | A | A | A | A | A | A | A | A | A | A | A | A | A | G | A | A | A | A | A | A | A | A | A | A | A | A | A | A | A | A | A | A | A | A | A | A | A | A | A | A | A | A | A | A | A | A | A | A | A | A |
| 785 | A | A | A | A | A | A | A | A | A | A | A | A | A | A | A | A | A | A | A | A | A | A | A | A | A | A | A | A | A | G | A | A | A | A | A | A | A | A | A | A | A | A | A | A | A | A | A | A | A | A | A | A | A | A | A | A | A | A |
| 810 | C | C | C | C | C | C | T | C | C | C | C | C | C | C | C | C | C | C | C | C | C | C | C | C | C | C | C | C | C | C | C | C | C | C | C | C | C | C | C | C | C | C | C | C | C | C | C | C | C | C | C | C | C | C | C | C | C | C |
| 831 | G | G | G | G | G | T | T | G | G | G | G | G | G | G | G | G | G | G | T | G | G | G | G | G | G | G | G | G | G | G | T | T | G | G | G | G | G | G | G | G | G | G | G | G | G | G | G | G | G | G | G | G | T | G | G | G | G | G |
| 836 | A | A | A | A | A | A | A | A | A | A | A | A | G | A | A | A | A | A | A | G | A | A | A | A | A | A | A | A | A | A | A | A | A | A | A | A | A | A | A | A | A | A | A | A | A | A | A | A | A | A | A | A | A | A | A | A | A | A |
| 885 | G | G | G | G | G | G | G | G | G | G | G | G | G | G | G | G | G | G | G | G | G | G | G | G | G | G | G | G | G | G | G | G | G | G | G | G | G | G | A | G | G | G | G | G | G | G | G | G | G | G | G | G | G | G | G | G | G | G |
| 891 | T | T | T | T | T | T | T | T | T | T | T | T | T | T | T | T | T | A | T | T | T | T | T | T | T | T | T | T | T | T | T | T | T | T | T | T | T | T | T | T | T | T | T | T | T | T | T | T | T | T | T | T | T | T | T | T | T | T |
| 903 | A | A | A | A | A | A | A | A | A | A | A | A | A | A | A | A | A | A | G | A | A | A | A | A | A | A | A | A | A | A | A | A | A | A | A | A | A | A | A | A | A | A | A | A | A | A | A | A | A | A | A | A | A | A | A | A | A | A |
| 931 | A | A | A | A | A | A | A | A | A | A | G | A | A | A | A | A | A | A | A | A | A | A | A | A | A | A | A | A | A | A | A | A | A | A | A | A | A | A | A | A | A | A | A | A | A | A | A | A | A | A | A | A | A | A | A | A | A | A |
| 939 | A | A | A | A | A | A | A | A | A | A | A | A | A | A | A | A | A | A | A | A | A | A | A | A | A | A | A | A | A | A | A | A | A | A | A | A | G | A | A | A | A | A | A | A | A | A | A | A | G | A | A | A | A | A | A | A | A | A |
| 951 | T | T | T | T | T | T | T | T | T | T | T | T | T | T | T | T | T | C | T | T | T | T | T | T | T | T | T | T | T | T | T | T | T | T | T | T | T | T | T | T | T | T | T | T | T | T | T | T | T | T | T | T | T | T | T | T | T | T |
| 960 | G | G | G | G | G | G | G | G | G | G | G | G | G | G | G | G | G | G | G | G | G | G | G | G | G | G | G | G | G | G | G | G | G | G | G | G | G | G | G | G | G | G | G | G | G | G | G | G | G | A | A | G | G | G | G | G | G | G |
| 965 | T | T | T | T | T | T | T | T | T | T | T | T | T | T | T | T | T | T | T | T | T | T | T | T | T | T | T | T | T | T | T | T | T | T | T | T | T | T | T | T | T | T | G | T | T | T | T | T | T | T | T | T | T | T | T | T | T | T |
| 969 | G | G | G | G | G | G | G | G | G | G | G | G | G | G | G | G | G | G | G | G | G | G | G | G | G | G | G | G | G | G | G | G | G | G | G | G | G | G | A | G | G | G | A | G | G | G | G | G | G | G | G | G | G | G | G | G | G | G |
| 994 | C | C | C | C | C | C | C | C | C | C | C | C | C | C | C | C | C | C | C | C | C | C | C | C | C | T | C | C | C | C | C | C | C | C | C | C | C | C | C | C | C | C | C | C | C | C | C | C | C | C | C | C | C | C | C | C | C | C |
| 999 | G | G | G | G | G | G | G | G | G | G | G | G | G | G | G | G | G | G | G | G | A | G | G | G | G | G | G | G | G | G | G | G | G | G | G | G | G | G | G | G | G | G | G | G | G | G | G | G | G | G | G | G | G | G | G | G | G | C |
| 1014 | T | C | C | C | C | C | C | C | C | C | T | C | C | C | C | C | C | C | C | C | C | C | C | C | T | T | T | C | C | C | C | C | C | T | C | T | T | T | C | T | T | C | C | C | T | T | C | T | T | C | C | C | C | C | C | C | C | T |
| 1088 | G | G | G | G | G | G | G | G | G | G | G | G | G | G | G | G | G | G | G | G | G | G | G | G | G | G | G | G | G | G | G | G | G | G | G | A | G | G | G | G | G | G | G | G | G | G | G | G | G | G | G | G | G | G | G | G | G | G |
| 1128 | C | C | C | C | A | C | C | C | C | C | C | C | C | A | C | C | C | C | C | C | C | C | C | C | C | C | C | C | C | C | C | C | C | C | C | C | C | C | C | C | C | C | C | C | C | C | C | C | C | C | C | C | C | C | C | C | C | C |
| 1131 | A | A | A | A | A | A | A | A | A | A | A | A | G | A | A | A | A | A | A | G | A | A | A | A | A | A | A | A | A | A | A | A | A | A | A | A | A | A | A | A | A | A | A | A | A | A | A | A | A | A | A | A | A | A | A | A | A | A |
| 1135 | G | G | G | G | G | G | G | G | G | G | G | G | G | G | G | G | G | G | G | G | G | G | G | G | G | G | G | G | G | G | G | G | G | G | G | G | G | G | A | G | G | G | A | G | G | G | G | G | G | G | G | G | G | G | G | G | G | G |
| 1136 | T | T | T | T | T | T | T | T | T | T | T | T | T | T | T | T | T | T | T | T | T | T | T | T | T | T | T | T | T | T | T | T | C | T | T | T | T | T | T | T | T | T | T | T | T | T | T | T | T | T | T | T | T | T | T | T | T | T |

| | | | | | | | | | | | | | | | | | | | | | | | | | | | | | | | | | | | | | | | | | | | | | | | | | | | | | | | | | | |
|---|---|---|---|---|---|---|---|---|---|---|---|---|---|---|---|---|---|---|---|---|---|---|---|---|---|---|---|---|---|---|---|---|---|---|---|---|---|---|---|---|---|---|---|---|---|---|---|---|---|---|---|---|---|---|---|---|---|---|
| 1140 | C | C | C | C | C | C | C | C | C | C | C | C | C | C | C | T | C | C | C | C | C | C | C | C | C | C | C | C | C | C | C | C | C | C | C | C | C | C | C | C | C | C | C | C | C | C | C | C | C | C | C | C | C | C | C | C | C | C |
| 1197 | C | C | C | C | C | C | C | T | C | T | C | C | C | C | T | T | C | T | T | C | C | T | T | C | C | C | C | C | T | C | C | T | C | C | C | C | C | C | T | C | C | C | T | C | C | C | C | C | C | C | C | C | C | C | C | C | C | C |
| 1218 | A | A | A | A | A | A | A | A | A | A | A | A | A | A | A | A | A | A | A | A | A | A | A | A | A | A | A | A | A | A | A | A | A | A | A | A | A | A | A | A | A | A | A | A | A | A | A | A | A | G | G | A | A | A | A | A | A | A |
| 1269 | G | G | G | G | G | G | G | G | G | G | G | G | G | G | G | G | G | C | G | G | G | G | G | G | G | G | G | G | G | G | G | G | G | G | G | G | G | G | G | G | G | G | G | G | G | G | G | G | G | G | G | G | G | G | G | G | G | G |
| 1315 | A | A | A | A | A | A | A | A | A | A | A | A | A | A | A | A | G | A | A | A | A | A | A | A | A | A | A | A | A | A | A | A | A | A | A | A | A | A | A | A | A | A | A | A | A | A | A | A | A | A | A | A | A | A | A | A | A | A |
| 1322 | C | C | C | C | C | C | C | C | C | C | C | C | C | C | C | C | C | T | C | C | C | C | C | C | C | C | C | C | C | C | C | C | C | C | C | C | C | C | C | C | C | C | C | C | C | C | C | C | C | C | C | C | C | C | C | C | C | C |
| 1329 | T | T | T | T | T | T | T | T | T | T | T | T | T | T | T | T | T | T | T | T | T | T | T | T | T | T | G | T | T | T | T | T | T | T | T | G | T | T | T | T | T | T | T | T | T | G | T | T | T | T | T | T | T | T | T | T | T | T |
| 1463 | T | T | T | T | T | T | T | T | T | T | T | T | G | T | T | T | T | T | T | G | T | T | T | T | T | T | T | T | T | T | T | T | T | T | T | T | T | T | T | T | T | T | T | T | T | T | T | T | T | T | T | T | T | T | T | T | T | T |
| 1465 | G | G | G | G | G | G | A | G | G | G | G | G | G | G | G | G | G | G | G | G | G | G | G | G | G | G | G | G | G | G | G | G | G | G | G | G | G | G | G | G | G | G | G | G | G | G | G | G | G | G | G | G | G | G | G | G | G | G |
| 1532 | T | T | T | T | T | T | T | T | T | T | T | T | T | T | T | T | T | C | T | T | T | T | T | T | T | T | T | T | T | T | T | T | T | T | T | T | T | T | T | T | T | T | T | T | T | T | T | T | T | T | T | T | T | T | T | T | T | T |
| 1544 | A | G | G | G | G | G | G | G | G | G | A | G | G | G | G | G | G | G | G | G | G | G | G | G | A | A | A | G | G | G | G | G | G | A | G | A | A | A | G | A | A | G | G | G | A | A | G | A | A | G | G | G | G | G | A | G | G | A |
| 1553 | A | A | A | A | A | A | A | A | A | A | A | A | A | A | A | A | A | A | G | A | A | A | A | A | A | A | A | A | A | A | A | A | A | A | A | A | A | A | A | A | A | A | A | A | A | A | A | A | A | A | A | A | A | A | A | A | A | A |
| 1567 | G | G | G | G | G | T | T | G | G | G | G | G | T | G | G | G | G | G | G | T | G | G | G | G | G | G | G | G | G | G | T | G | T | G | G | G | G | G | G | G | G | G | G | G | G | G | G | G | G | G | G | G | G | G | G | G | G | G |
| 1595 | T | T | T | T | T | T | T | T | T | T | T | T | T | T | T | T | T | T | C | T | T | T | T | T | T | T | T | T | T | T | T | T | T | T | T | T | T | T | T | T | T | T | T | T | T | T | T | T | T | T | T | T | T | T | T | T | T | T |
| 1607 | G | A | A | A | G | G | G | G | G | G | G | A | G | G | G | G | G | G | G | G | G | G | G | G | G | G | G | G | G | G | G | G | G | G | G | G | G | G | G | G | G | G | G | G | G | G | G | G | G | G | G | G | G | G | G | G | G | G |
| 1608 | C | C | C | C | C | C | C | C | C | C | C | C | C | C | C | C | C | C | C | C | C | C | C | C | C | C | C | C | C | C | C | C | C | C | C | C | C | C | C | C | C | C | C | C | C | C | C | T | C | C | C | C | C | C | C | C | C | C |
| 1609 | C | C | C | C | C | C | C | C | C | C | C | C | C | C | C | C | C | T | C | C | C | C | C | C | C | C | C | C | C | C | C | C | C | C | C | C | C | C | C | C | C | C | C | C | C | C | C | C | C | C | C | C | C | C | C | C | C | C |
| 1623 | G | G | G | G | G | C | C | G | G | G | G | G | G | G | G | G | G | G | G | G | G | G | G | G | G | G | G | G | G | G | C | G | C | G | G | G | G | G | G | G | G | G | G | G | G | G | G | G | G | G | G | G | G | G | G | G | G | G |
| 1630 | G | G | G | G | G | C | G | G | G | G | G | G | G | G | G | G | G | G | G | G | G | G | G | G | G | G | G | G | G | G | G | G | G | G | G | G | G | G | G | G | G | G | G | G | G | G | G | G | G | G | G | G | G | G | G | G | G | G |
| 1651 | C | C | C | C | C | C | C | C | C | C | C | T | C | C | C | C | C | C | C | C | C | C | C | C | C | C | C | C | C | C | C | C | C | C | C | C | C | C | C | C | C | C | C | C | C | C | C | C | C | C | C | C | C | C | C | C | C | C |
| 1658 | C | C | C | C | C | C | C | C | C | C | C | C | C | C | C | C | C | C | T | C | C | C | C | C | C | C | C | C | C | C | C | C | C | C | C | C | C | C | C | C | C | C | C | C | C | C | C | C | C | C | C | C | C | C | C | C | C | C |
| 1675 | C | C | C | C | C | C | C | C | C | C | C | C | C | C | C | C | C | C | C | C | C | C | C | C | C | C | C | C | C | C | C | C | C | C | C | C | C | C | C | C | C | C | C | C | C | C | C | C | C | T | T | C | C | C | C | C | C | C |
| 1706 | C | C | C | C | C | C | C | T | C | C | C | C | C | C | C | C | C | T | C | C | C | C | C | C | C | C | C | C | C | C | C | C | C | C | C | C | C | C | C | C | C | C | C | C | C | C | C | C | C | C | C | C | C | C | C | C | C | C |
| 1713 | G | G | G | G | T | T | T | T | G | T | G | G | T | T | T | T | G | T | T | T | G | T | T | G | G | G | G | G | T | G | T | T | T | G | G | G | G | G | T | G | G | G | T | G | G | G | G | G | G | T | T | G | G | G | G | G | G | G |
| 1718 | T | T | T | T | T | T | T | T | T | T | T | T | T | T | T | T | T | T | C | T | T | T | T | T | T | T | T | T | T | T | T | T | T | T | T | T | T | T | T | T | T | T | T | T | T | T | T | T | T | T | T | T | T | T | T | T | T | T |

| | | | | | | | | | | | | | | | | | | | | | | | | | | | | | | | | | | | | | | | | | | | | | | | | | | | | | | | | | | |
|---|---|---|---|---|---|---|---|---|---|---|---|---|---|---|---|---|---|---|---|---|---|---|---|---|---|---|---|---|---|---|---|---|---|---|---|---|---|---|---|---|---|---|---|---|---|---|---|---|---|---|---|---|---|---|---|---|---|---|
| 1729 | A | A | A | A | G | G | G | G | A | G | A | A | G | G | G | G | A | G | G | G | A | G | G | A | A | A | A | A | G | A | G | G | G | A | A | A | A | A | G | A | A | A | G | A | A | A | A | A | A | G | G | A | A | A | A | A | A | A |
| 1766 | C | C | C | C | T | C | C | C | C | C | C | C | C | T | C | C | C | C | C | C | C | C | C | C | C | C | C | C | A | C | C | C | C | C | C | C | C | C | C | C | C | C | C | C | C | C | C | C | C | C | C | C | C | C | C | C | C | C |
| 1810 | C | C | C | C | C | C | C | C | C | C | C | C | T | C | C | C | C | C | C | T | C | C | C | C | C | C | C | C | C | C | C | C | C | C | C | C | C | C | C | C | C | C | C | C | C | C | C | C | C | C | C | C | C | C | C | C | C | C |
| 1811 | G | G | G | G | G | G | G | G | G | G | G | G | G | G | G | G | G | G | G | G | G | G | G | G | A | G | G | G | G | G | G | G | G | G | G | G | G | G | G | G | G | G | G | G | G | G | G | G | G | G | G | G | G | G | G | G | G | G |
| 1832 | C | C | C | C | C | C | C | C | C | C | C | C | C | C | C | C | C | C | C | C | C | C | C | G | C | C | C | C | C | C | C | C | C | C | C | C | C | C | C | C | C | C | C | C | C | C | C | C | C | C | C | C | C | C | C | C | C | C |
| 1842 | C | C | C | C | G | C | C | C | C | C | C | C | C | C | C | C | C | C | C | C | C | C | C | C | C | C | C | C | C | C | C | C | C | C | C | C | C | C | C | C | C | C | C | C | C | C | C | C | C | C | C | C | C | C | C | C | C | C |
| 1874 | T | T | T | T | A | A | A | A | T | A | T | T | A | A | A | A | T | A | A | A | T | A | A | T | T | T | T | T | A | T | A | A | A | T | T | T | T | T | A | T | T | T | A | T | T | T | T | T | T | A | A | T | T | T | T | T | T | T |
| 1903 | A | A | A | A | A | A | A | A | A | A | A | A | A | A | A | A | T | A | A | A | A | A | A | A | A | A | A | A | A | A | A | A | A | A | A | A | A | A | A | A | A | A | A | A | A | A | A | A | A | A | A | A | A | A | A | A | A | A |
| 1935 | A | A | A | A | A | A | A | A | A | A | A | A | A | A | G | A | A | A | A | A | A | A | A | A | A | A | A | A | A | A | A | A | A | A | A | A | A | A | A | A | A | A | A | A | A | A | A | A | A | A | A | A | A | A | A | A | A | A |
| 1968 | G | G | G | G | G | G | G | G | G | G | G | G | G | G | G | G | G | G | G | G | G | G | G | G | G | G | G | G | A | G | G | G | G | G | G | G | G | G | G | G | G | G | G | G | G | G | G | G | G | G | G | G | G | G | G | G | G | G |
| 1972 | G | G | G | G | G | G | G | G | G | G | G | G | A | G | G | G | G | G | G | A | G | G | G | G | G | G | G | G | G | G | G | G | G | G | G | G | G | G | G | G | G | G | G | G | G | G | G | G | G | G | G | G | G | G | G | G | G | G |
| 2005 | T | G | G | G | G | G | G | G | G | G | T | G | G | G | G | G | G | G | G | G | G | G | G | G | T | T | T | G | G | G | G | G | G | T | G | T | T | T | G | T | T | G | G | G | T | T | G | T | T | G | G | G | G | T | G | G | G | T |
| 2009 | A | G | G | G | G | G | G | G | G | G | A | G | G | G | G | G | G | G | G | G | G | G | G | G | A | A | A | G | G | G | G | G | G | A | G | A | A | A | G | A | A | G | G | G | A | A | G | A | A | G | G | G | G | G | G | G | G | A |
| 2073 | T | T | T | T | T | T | T | T | T | T | T | T | T | T | T | T | T | T | T | T | T | T | T | T | T | T | T | T | T | T | T | T | T | T | T | T | T | T | T | T | T | T | T | T | T | T | T | T | T | A | A | T | T | T | T | T | T | T |
| 2082 | A | A | A | A | A | A | A | A | A | A | A | A | A | A | A | C | A | A | A | A | A | A | A | A | A | A | A | A | A | A | A | A | A | A | A | A | A | A | A | A | A | A | A | A | A | A | A | A | A | A | A | A | A | A | A | A | A | A |
| 2097 | A | A | A | A | A | A | A | A | A | A | A | A | A | A | A | A | A | A | T | A | A | A | A | A | A | A | A | A | A | A | A | A | A | A | A | A | A | A | A | A | A | A | A | A | A | A | A | A | A | A | A | A | A | A | A | A | A | A |
| 2118 | A | A | A | A | A | A | A | A | A | A | G | A | A | A | A | A | A | A | A | A | A | A | A | A | A | A | A | A | A | A | A | A | A | A | A | A | A | A | A | A | A | A | A | A | A | A | A | A | A | A | A | A | A | A | A | A | A | A |
| 2132 | G | G | G | G | G | G | G | G | G | G | G | G | G | G | G | G | G | G | G | G | G | G | G | G | G | G | G | G | G | G | G | G | G | G | G | G | G | G | G | G | G | G | G | G | G | G | G | G | G | A | A | G | G | G | G | G | G | G |
| 2133 | C | C | C | C | C | C | C | C | C | C | C | C | T | C | C | C | C | C | C | T | C | C | C | C | C | C | C | C | C | C | T | C | C | C | C | C | C | C | C | C | C | C | C | C | C | C | C | C | C | C | C | C | C | C | C | C | C | C |
| 2175 | C | C | C | C | C | C | C | C | C | C | C | C | C | C | C | C | T | C | C | C | C | C | C | C | C | C | C | C | C | C | C | C | C | C | C | C | C | C | C | C | C | C | C | C | C | C | C | C | C | C | C | C | C | C | C | C | C | C |
| 2197 | C | C | C | C | C | C | C | C | C | T | C | C | C | C | C | C | C | C | C | C | C | C | C | C | C | C | C | C | C | C | C | C | C | C | C | C | C | C | T | C | C | C | T | C | C | C | C | C | C | C | C | C | C | C | C | C | C | C |
| 2209 | T | C | C | C | C | C | C | C | C | C | T | C | C | C | C | C | C | C | C | C | C | C | C | C | T | T | T | C | C | C | C | C | C | T | C | T | T | T | C | T | T | C | C | C | T | T | C | T | T | C | C | C | C | C | C | C | C | T |
| 2211 | G | G | G | G | G | G | G | G | G | G | G | G | G | G | G | G | G | G | G | G | G | G | G | G | G | G | G | G | G | G | G | G | G | G | G | G | G | G | G | G | G | G | G | G | G | G | G | G | G | A | A | G | G | G | G | G | G | G |
| 2217 | C | C | C | C | C | C | C | C | C | C | C | C | C | C | C | C | C | C | C | C | C | C | C | C | C | C | C | C | C | C | C | C | C | C | C | C | C | C | C | C | C | C | C | C | C | G | C | C | C | C | C | C | C | C | C | C | C | C |
| 2252 | A | T | T | A | A | A | A | A | A | A | A | A | A | A | A | A | A | A | A | A | A | A | A | A | A | A | A | A | A | A | A | A | A | A | A | A | A | A | A | A | A | A | A | A | A | A | A | A | A | A | A | A | A | A | A | A | A | A |

| | | | | | | | | | | | | | | | | | | | | | | | | | | | | | | | | | | | | | | | | | | | | | | | | | | | | | | | | | | |
|---|---|---|---|---|---|---|---|---|---|---|---|---|---|---|---|---|---|---|---|---|---|---|---|---|---|---|---|---|---|---|---|---|---|---|---|---|---|---|---|---|---|---|---|---|---|---|---|---|---|---|---|---|---|---|---|---|---|---|
| 2309 | T | T | T | T | T | T | T | T | T | T | T | T | A | T | T | T | T | T | T | A | T | T | T | T | T | T | T | T | T | T | T | T | T | T | T | T | T | T | T | T | T | T | T | T | T | T | T | T | T | T | T | T | T | T | T | T | T | T |
| 2350 | T | T | T | T | T | A | A | T | T | T | T | T | T | T | T | T | T | T | T | T | T | T | T | T | T | T | T | T | T | T | A | T | T | T | T | T | T | T | T | T | T | T | T | T | T | T | T | T | T | T | T | T | T | T | T | T | T | T |
| 2363 | C | C | C | C | C | C | C | C | C | T | C | C | C | C | C | C | C | C | C | C | C | C | C | C | C | C | C | C | C | C | C | C | C | C | C | C | C | C | C | C | C | C | C | C | C | C | C | C | C | C | C | C | C | C | C | C | C | C |
| 2396 | A | A | A | A | G | A | A | A | A | A | A | A | A | G | A | A | A | A | A | A | A | A | A | A | A | A | A | A | A | A | A | A | A | A | A | A | A | A | A | A | A | A | A | A | A | A | A | A | A | A | A | A | A | A | A | A | A | A |
| 2436 | T | T | T | T | T | T | T | T | T | T | T | T | T | T | T | T | C | T | T | T | T | T | T | T | T | T | T | T | T | T | T | T | T | T | T | T | T | T | T | T | T | T | T | T | T | T | T | T | T | T | T | T | T | T | T | T | T | T |
| 2444 | A | A | A | A | T | A | A | T | A | T | A | A | A | T | A | T | A | T | T | A | A | T | A | A | A | A | A | A | T | A | A | T | T | A | A | A | A | A | T | A | A | A | T | A | A | A | A | A | A | A | A | A | A | A | A | T | A | A |
| 2470 | G | A | A | A | G | G | G | G | G | G | G | A | G | G | G | G | G | G | G | G | G | G | G | G | G | G | G | G | G | G | G | G | G | G | G | G | G | G | G | G | G | G | G | G | G | G | G | G | G | G | G | G | G | G | G | G | G | G |
| 2523 | G | G | G | G | G | G | G | G | G | G | G | G | G | G | G | G | G | G | G | G | G | G | G | G | G | G | G | G | G | G | G | G | G | G | G | G | G | G | G | G | A | G | G | G | G | G | G | G | G | G | G | G | G | G | G | G | G | G |
| 2551 | G | G | G | G | G | G | G | G | G | G | G | G | G | A | G | G | G | G | G | G | G | G | G | G | G | G | G | G | G | G | G | G | G | G | G | G | A | G | G | A | G | G | G | G | G | G | G | G | A | G | G | G | G | G | G | G | G | G |
| 2552 | T | T | T | T | T | T | T | T | T | T | T | T | A | T | T | T | T | T | T | A | T | T | T | T | T | T | T | T | T | T | T | T | T | T | T | T | T | T | T | T | T | T | T | T | T | T | T | T | T | T | T | T | T | T | T | T | T | T |
| 2555 | C | C | C | C | C | C | C | C | C | C | C | C | C | C | C | C | C | C | C | C | C | C | C | C | C | C | C | C | C | C | T | C | C | C | C | C | C | C | C | C | C | C | C | C | C | C | C | C | C | C | C | C | C | C | C | C | C | C |
| 2566 | G | G | G | G | G | G | G | C | G | C | G | G | G | G | G | C | G | C | C | G | G | C | G | G | G | G | G | G | C | G | G | C | C | G | G | G | G | G | C | G | G | G | C | G | G | G | G | G | G | A | G | G | G | G | G | C | G | G |
| 2571 | G | G | G | G | G | G | G | G | G | G | G | G | G | G | G | G | G | G | C | G | G | G | G | G | G | G | G | G | G | G | G | C | G | G | G | G | G | G | G | G | G | G | G | G | G | G | G | G | G | G | G | G | G | G | G | G | G | G |
| 2575 | T | T | T | T | T | C | C | T | T | T | T | T | T | T | T | T | T | T | T | T | T | T | T | T | T | T | T | T | T | T | C | T | T | T | T | T | T | T | T | T | T | T | T | T | T | T | T | T | T | T | T | T | T | T | T | T | T | T |
| 2680 | A | A | A | A | G | G | G | G | A | G | A | A | G | G | A | G | A | G | G | G | A | G | A | A | A | A | A | A | G | A | G | G | G | A | A | A | A | A | G | A | A | A | G | A | A | A | A | A | A | G | G | A | A | A | A | G | A | A |
| 2687 | A | A | A | A | A | A | A | A | A | A | A | A | A | A | A | A | A | A | A | A | A | G | A | A | A | A | A | A | A | A | A | A | A | A | A | A | A | A | A | A | A | A | A | A | A | A | A | A | A | A | A | A | A | A | A | G | A | A |
| 2695 | G | G | G | G | G | G | G | G | G | G | G | G | G | G | G | G | G | G | G | G | G | G | G | G | G | G | G | G | G | G | G | G | G | G | G | G | G | A | G | G | G | G | G | G | G | G | G | G | G | G | G | G | G | G | G | G | G | G |
| 2697 | G | G | G | G | G | G | G | G | G | G | G | G | G | G | T | G | G | G | G | G | G | G | G | G | G | G | G | G | G | G | G | G | G | G | G | G | G | G | G | G | G | G | G | G | G | G | G | G | G | G | G | G | G | G | G | G | G | G |
| 2699 | G | G | G | G | A | G | G | G | G | G | G | G | G | A | G | G | G | G | G | G | G | G | G | G | G | G | G | G | G | G | G | G | G | G | G | G | G | G | G | G | G | G | G | G | G | G | G | G | G | G | G | G | G | G | G | G | G | G |
| 2712 | C | C | C | C | C | C | C | C | C | C | C | C | C | C | C | C | C | C | C | C | C | C | C | C | C | C | C | C | C | C | C | C | C | C | C | C | C | C | C | C | C | C | C | C | C | C | C | C | C | T | T | C | C | C | C | C | C | C |
| 2714 | T | T | T | T | T | T | T | T | T | T | T | T | T | T | T | T | T | C | T | T | T | T | T | T | T | T | T | T | T | T | T | T | T | T | T | T | T | T | T | T | T | T | T | T | T | T | T | T | T | T | T | T | T | T | T | T | T | T |
| 2739 | C | C | C | C | C | A | A | C | C | C | C | C | C | C | C | C | C | C | C | C | C | C | C | C | C | C | C | C | C | C | A | C | C | C | C | C | C | C | C | C | C | C | C | C | C | C | C | C | C | C | C | A | C | C | C | C | C | C |
| 2760 | A | A | A | A | A | A | A | A | A | A | A | A | A | A | A | A | A | G | A | A | A | A | A | A | A | A | A | A | A | A | A | A | A | A | A | A | A | A | A | A | A | A | A | A | A | A | A | A | A | A | A | A | A | A | A | A | A | A |

Table S5. Blast resistance spectra of *Pid3* and Hap_6 for 125 *M. oryzae* isolates

| Isolates | TP309 | Hap_6 | Pid3 | Isolates | TP309 | Hap_6 | Pid3 | Isolates | TP309 | Hap_6 | Pid3 |
|---|---|---|---|---|---|---|---|---|---|---|---|
| 02-11-35-3 | S | S | S | 10-120-14-1 | S | S | S | 10-25-2-1 | S | S | S |
| 03-10-66-1 | S | R | R | 10-120-16-1 | S | S | S | 10-31-2-1 | S | S | S |
| 03-10-67-1 | S | R | R | 10-120-21-2 | S | S | S | 10-32-1-1 | S | S | S |
| 03-10-76-3 | S | R | R | 10-120-25-2 | S | S | S | 10-32-2-1 | S | R | R |
| 03-10-77-1 | S | R | R | 10-120-27-2 | S | S | S | 10-32-3-2 | S | S | S |
| 03-11-37-1 | S | S | S | 10-120-72-1 | S | S | S | 10-38-1-1 | S | S | S |
| 04-1-10-1 | S | S | S | 10-120-75-1 | S | S | S | 10-43-1-1 | S | S | S |
| 04-12-8-1 | S | S | S | 10-120-76-2 | S | S | S | 10-43-5-2 | S | S | S |
| 04-8-2-1 | S | S | S | 10-120-77-1 | R | R | R | 10-47-11-1 | S | S | S |
| 04-9-7-1 | S | S | S | 10-12-1-1 | S | S | S | 10-47-11-2 | S | S | S |
| 05-129-2-1 | S | S | S | 10-12-2-1 | S | S | S | 10-47-12-1 | S | S | S |
| 05-2-7-3 | S | S | S | 10-12-2-3 | S | S | S | 10-47-12-2 | S | S | S |
| 05-3-17-1 | S | S | S | 10-12-3-2 | S | S | S | 10-47-13-1 | S | S | S |
| 06-45-1-3 | S | R | R | 10-12-3-4 | S | S | S | 10-47-14-1 | S | R | R |
| 07-21-1-1 | S | R | R | 10-12-34-1 | S | S | S | 10-47-15-1 | S | S | S |
| 07-24-1-1 | S | S | S | 10-12-35-2 | S | S | S | 10-47-15-2 | S | R | R |
| 07-26-22 | S | R | R | 10-12-36-1 | S | S | S | 10-47-16-1 | S | R | R |
| 07-31-1-2 | S | R | R | 10-12-37-2 | S | S | S | 10-47-16-2 | S | R | R |
| 07-38-2-1 | S | R | R | 10-12-4-4 | S | S | S | 10-47-17-1 | S | R | R |
| 07-55-1-1 | S | R | R | 10-128-1-1 | S | S | S | 10-47-17-3 | S | S | S |
| 08-115-3-1 | S | S | S | 10-128-2-1 | S | S | S | 10-47-2-1 | S | S | S |
| 08-115-4-1 | S | S | S | 10-128-5-2 | S | S | S | 10-47-3-2 | S | S | S |
| 08-128-15-1 | R | R | R | 10-135-1-1 | S | S | S | 10-47-7-1 | S | S | S |
| 08-32-4-1 | S | S | S | 10-135-2-2 | S | S | S | 10-47-9-1 | S | S | S |
| 08-32-6-1 | S | S | S | 10-135-3-1 | S | S | S | 10-47-9-2 | S | R | R |
| 09-127-6-1 | S | S | S | 10-135-5-2 | S | S | S | 10-48-1-1 | S | S | S |
| 09-135-7-1 | S | S | S | 10-14-1-1 | S | S | S | 10-62-2-1 | S | S | S |
| 09-7-3-1 | S | R | R | 10-14-3-2 | S | R | R | 10-62-3-1 | S | S | S |
| 09-86-1-2 | S | S | S | 10-145-1 | S | S | S | 91-65-1 | S | S | S |
| 09-87-2-1 | R | R | R | 10-14-5-1 | S | S | S | 97-27-2 | S | R | R |
| 09-9-1-1 | S | S | S | 10-145-1-2 | S | S | S | 99-20-2 | S | R | R |
| 10-117-1-1 | S | S | S | 10-145-2-1 | S | S | S | 99-26-1 | S | S | S |
| 10-117-11-1 | S | S | S | 10-145-4-1 | S | S | S | 99-26-2 | S | R | R |
| 10-117-13-2 | S | S | S | 10-15-2-1 | S | S | S | CH43 | S | S | S |
| 10-117-15-2 | S | S | S | 10-19-1-1 | S | S | S | CH45 | S | S | S |
| 10-117-17-1 | S | S | S | 10-21-1-1 | S | S | S | CH704 | S | S | S |
| 10-117-19-1 | S | S | S | 10-21-11-1 | S | S | S | CH706 | S | S | S |
| 10-117-3-2 | S | S | S | 10-21-3-1 | S | S | S | Chuan26 | S | S | S |
| 10-117-7-1 | S | S | S | 10-21-6-1 | S | R | R | Chuan36 | S | S | S |
| 10-117-9-1 | S | S | S | 10-21-9-1 | S | S | S | JS2001-108-1 | S | R | R |
| 10-120-13-1 | S | S | S | 10-25-1-1 | S | S | S | Y34 | S | R | R |
| ZB13 | S | S | S | ZB15 | S | S | S | | | | |

Table S6. Comparation of resistance frequency of Pid3-I2/Hap_6 and *Pid3*

| **Transgenic lines** | **TP309** | ***Pid3-I2/Hap_6*** | ***Pid3*** |
|---|---|---|---|
| Total *M. oryzae* strains | 125 | 125 | 125 |
| Compatible strains | 122 | 97 | 97 |
| Incompatible strains | 3 | 28 | 28 |
| Resistance frequency | 2.4% | 22.4% | 22.4% |

Table S7. Nucleotide polymorphism of *Pid3* in 1407 hybrid rice varieties

| Haplotype | NO. | 458 | 477 | 525 | 537 | 610 | 1014 | 1544 | 1811 | 1874 | 2209 | 2444 |
|---|---|---|---|---|---|---|---|---|---|---|---|---|
| Hap_6 | | C | G | C | A | G | C | G | G | T | C | A |
| Hap_H1* | 1392 | C | G | C | A | G | C | G | G | T | C | A |
| Hap_H2* | 1 | C | A | T | G | G | C | G | G | A | C | T |
| Hap_H3* | 13 | T | A | T | G | A | T | A | G | T | T | A |
| Hap_H4* | 1 | T | A | T | G | A | T | A | A | T | T | A |

No., Numbers of rice accessions belonged to specific haplotype. Hap_H1 was identical to Hap_6/15/21/22/23/25/26/29; Hap_H2 was identical to Hap_1/2/11/19/20; Hap_H3 was identical to Hap_7/9/24/30/31/32/33/35/36/37/38/39/40; Hap_H14 was identical to Hap_8.
